# Pan-Genome Analysis of Transcriptional Regulation in Six Salmonella enterica Serovar Typhimurium Strains Reveals Their Different Regulatory Structures

**DOI:** 10.1128/msystems.00467-22

**Published:** 2022-11-01

**Authors:** Yuan Yuan, Yara Seif, Kevin Rychel, Reo Yoo, Siddharth Chauhan, Saugat Poudel, Tahani Al-bulushi, Bernhard O. Palsson, Anand V. Sastry

**Affiliations:** a Department of Bioengineering, University of California San Diego, La Jolla, California, USA; b Novo Nordisk Foundation Center for Biosustainability, Technical University of Denmark, Lyngby, Denmark; Vanderbilt University Medical Center

**Keywords:** *Salmonella*, ICA, pan-genomics, pan-genome, *Salmonella* Typhimurium, systems biology, transcriptomics, gene regulation

## Abstract

Establishing transcriptional regulatory networks (TRNs) in bacteria has been limited to well-characterized model strains. Using machine learning methods, we established the transcriptional regulatory networks of six Salmonella enterica serovar Typhimurium strains from their transcriptomes. By decomposing a compendia of RNA sequencing (RNA-seq) data with independent component analysis, we obtained 400 independently modulated sets of genes, called iModulons. We (i) performed pan-genome analysis of the phylogroup structure of *S*. Typhimurium and analyzed the iModulons against this background, (ii) revealed different genetic signatures in pathogenicity islands that explained phenotypes, (iii) discovered three transport iModulons linked to antibiotic resistance, (iv) described concerted responses to cationic antimicrobial peptides, and (v) uncovered new regulons. Thus, by combining pan-genome and transcriptomic analytics, we revealed variations in TRNs across six strains of serovar Typhimurium.

**IMPORTANCE** Salmonella enterica serovar Typhimurium is a pathogen involved in human nontyphoidal infections. Treating *S*. Typhimurium infections is difficult due to the species’s dynamic adaptation to its environment, which is dictated by a complex transcriptional regulatory network (TRN) that is different across strains. In this study, we describe the use of independent component analysis to characterize the differential TRNs across the *S*. Typhimurium pan-genome using a compendium of high-quality RNA-seq data. This approach provided unprecedented insights into the differences between regulation of key cellular functions and pathogenicity in the different strains. The study provides an impetus to initiate a large-scale effort to reveal the TRN differences between the major phylogroups of the pathogenic bacteria, which could fundamentally impact personalizing treatments of bacterial pathogens.

## INTRODUCTION

Salmonella enterica is one of the leading causes of foodborne illnesses globally (1), resulting in an estimated 93 million infected individuals per year, 1.35 million of which are in the United States (1, 2). While nontyphoidal Salmonella enterica is highly diverse (3), serovar Typhimurium is of particular interest because it has broad host specificity and poses serious challenges to public health, especially with the rise in its antibiotic resistance and the advent of new strains causing serious to life-threatening infections in sub-Saharan Africa (4). Infections caused by *S*. Typhimurium are difficult to combat for two primary reasons. First, *S*. Typhimurium contains a wide variety of strains with different genetic and phenotypic signatures. These strains vary in virulence, persistence, and response to diverse conditions and thus require different treatment methods. Second, *S*. Typhimurium strains use a set of intricate mechanisms to adapt to their host, develop drug resistance, and enhance virulence. These mechanisms are activated by the transcriptional regulatory networks (TRNs) that coordinate gene expression under a variety of different conditions, including antibiotic treatment, starvation, and stress. Well-characterized TRNs of the serovar as a whole and in individual strains would enable us to develop a better understanding of *S*. Typhimurium’s dynamic adaptations to environmental perturbations and to develop treatment options to improve clinical outcomes. Gaining a deeper understanding of the TRN of S. enterica Typhimurium therefore holds great importance for public health.

To date, efforts to characterize *S*. Typhimurium’s TRNs have mainly been confined to understanding the TRNs of individual strains. Prior studies comparing transcriptomic patterns across *S*. Typhimurium strains typically focused on observing differentially expressed genes of two strains (5). System-level investigations of TRNs, and particularly investigations across more strains and over the entire serovar, were hindered by inadequate data analytic methods.

The increasing number of sequenced genomes has empowered pan-genomic analysis to study the genetic diversity and composition of a species. A pan-genome contains all the genes in that species, and a core genome is defined as the genes shared between all strains in the species. Previous pan-genome analyses of Salmonella have identified unique genetic landscapes of different serovars and strains and demonstrated the potential to understand strain relationships and phenotype differences (6–8).

Recently, independent component analysis (ICA) has been successful in elucidating quantitative bacterial TRNs (9–11). ICA is a signal separation algorithm that deconvolutes mixed signals into their individual sources and determines their relative strengths (12). By applying ICA to bacterial transcriptomes, we can identify independently modulated sets of genes, called iModulons (individual source signals), and the activity level of an iModulon across different conditions (relative signal strength). Unlike regulons, which are derived from experimental data of transcription factors from DNA binding sites in a bottom-up fashion, iModulons are derived computationally from gene expression via a top-down approach. It has been applied to transcriptomic data sets of Escherichia coli, Bacillus subtilis, and Staphylococcus aureus and provided valuable insights into the global TRNs of these species (9–11). iModulon structures are available for a number of bacterial species (iModulonDB.org [13]).

In this study, we compute and analyze the iModulons of six *S*. Typhimurium strains and compare their gene composition and condition-dependent activity levels. We generated a large transcriptomic compendium by downloading all the publicly available RNA sequencing (RNA-seq) data of this serovar from the NCBI Sequence Read Archive (SRA) and compiled expression profiles of 534 high-quality samples. The large size and diversity of conditions in this data set make it a valuable starting point for machine learning of the TRN. In order to understand the serovar as a whole and simultaneously characterize individual strains, we performed a pan-genomic analysis on 506 *S*. Typhimurium strains and defined the *S*. Typhimurium core genome (3,886 genes) with 172 strains. We performed ICA on both the core genome and the individual strain genomes and obtained 400 robust iModulons in total. Many of these iModulons are highly consistent with known regulons, while others offer guidance for new discoveries. This study, for the first time, combines pan-genomic analysis to reveal the phylogroup structure of a subspecies and transcriptomic analysis to reveal strain-specific TRNs.

## RESULTS

### Pan-genomic analysis revealed the core *S*. Typhimurium genome.

To understand the genetic diversity of serovar Typhimurium, we collected 3,329 *S*. Typhimurium genomes from PATRIC (https://www.patricbrc.org/). After quality control (see Materials and Methods), we assembled an *S*. Typhimurium pan-genome across 506 strains containing 17,243 gene families.

**FIG 1:**
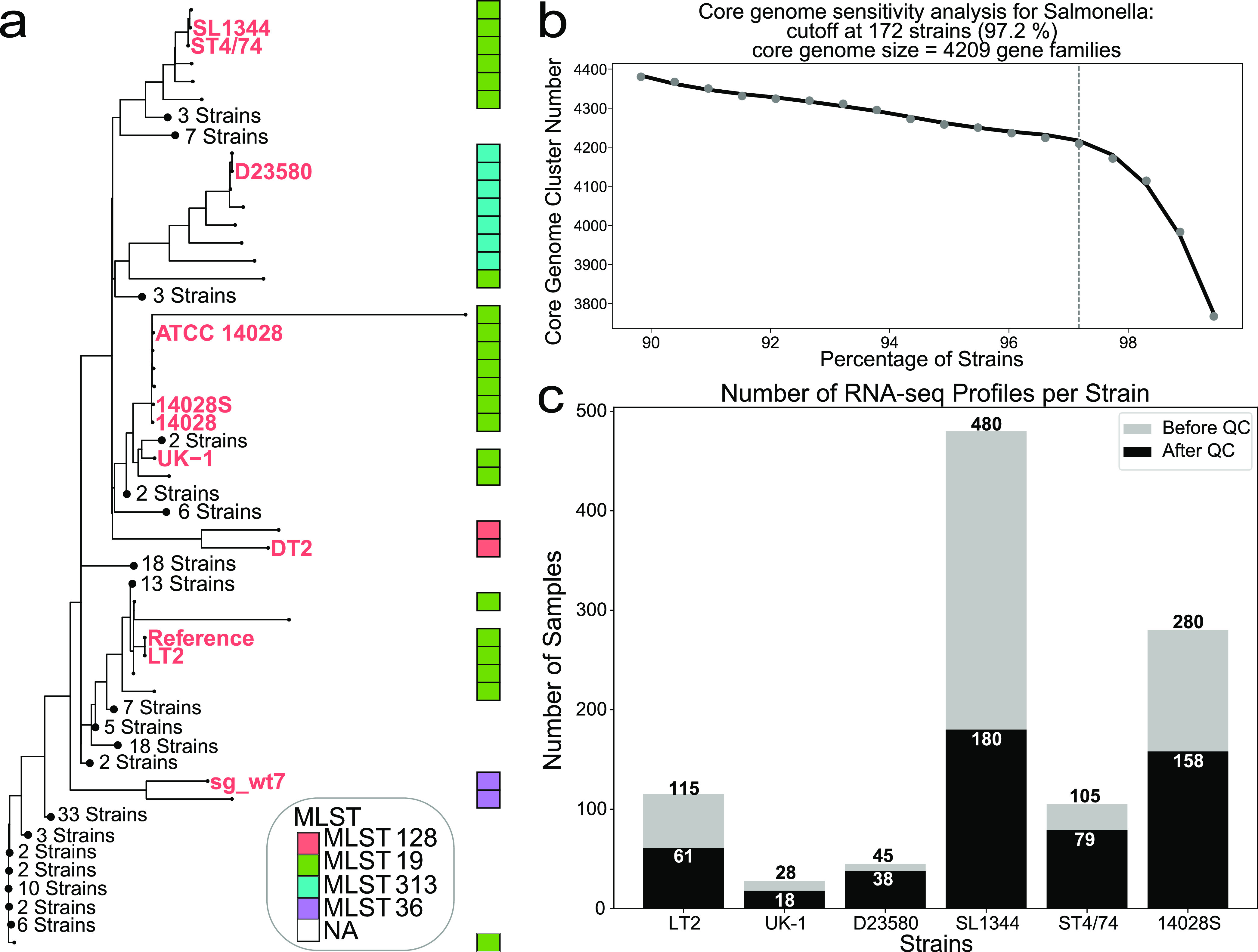
Data set overview, sensitivity analysis, and the phylogenetic tree. (a) Phylogenetic tree of 177 representative *S*. Typhimurium strains. Strains with names labeled in colors are strains with RNA-seq data in our compendium. Strains DT2 and sg_wt7 each had only four samples. Due to the small size of the data sets, these two strains were used to build the core genome but werenot included for individual ICA (see Materials and Methods). The multilocus sequence typing (MLST) information of each strain (gathered from PATRIC) is presented in the bar on the right. The complete tree with uncollapsed branches can be found in Fig. S1 in the supplemental material. Note that strains ATCC 14028, 14028S, and 14028 are considered the same strain in subsequent analyses (see Text S1 [Note 1]). (b) Sensitivity analysis for core genome definition. Many genomes from PATRIC are incomplete. To avoid losing too many gene families due to incomplete genomes, a sensitivity analysis was performed and a cutoff at 97.2% was chosen. Of 177 genomes, 172 were used to define the core genome, resulting in a final core genome size of 4,209 gene families. (c) The number of samples for each strain before and after the quality control (QC) pipeline. This bar chart shows the distribution of available RNA-seq data across six S. enterica Typhimurium strains. While SL1344 has the highest number of RNA-seq profiles, many failed QC because of the low number of reads mapped to coding sequences. The detailed quality control metrics can be found in Materials and Methods.

The phylogenetic tree of 177 representative strains (Fig. 1a) revealed that the eight strains in the transcriptomic compendium represent the spectrum of genetic diversity of *S*. Typhimurium. A majority of the 177 representative strains, including five of the eight strains in our transcriptomic compendium, belong to sequence type 19 (ST19), the most common sequence type of *S*. Typhimurium (14–16). Two ST19 strains (SL1344 and ST4/74) are closely related. In addition, D23580 is a derivative of ST4/74. Despite the small genetic difference of only 846 nucleotide variations, the two strains have different sequence types and differ significantly in transcriptional and phenotypic features (5). These phylogenetic relationships between strains in our compendium guide the comparisons of strain-specific transcriptomic differences.

10.1128/msystems.00467-22.1 FIG S1 Complete phylogenetic tree. The strains labeled in red are the strains with RNA-seq profiles. Download FIG S1, PDF file, 0.3 MB. Copyright © 2022 Yuan et al. 2022 Yuan et al. https://creativecommons.org/licenses/by/4.0/ This content is distributed under the terms of the Creative Commons Attribution 4.0 International license.

10.1128/msystems.00467-22.10 TEXT S1 Supplementary Notes. Download Text S1, DOCX file, 0.02 MB. Copyright © 2022 Yuan et al. 2022 Yuan et al. https://creativecommons.org/licenses/by/4.0/ This content is distributed under the terms of the Creative Commons Attribution 4.0 International license.

To mitigate the negative effect of the incomplete genome on the core genome size, a sensitivity analysis was performed on the 177 strains, resulting in a core genome spanning 172 strains and containing 4,209 gene families (Fig. 1b). For subsequent transcriptomic analysis of the core genome, we used a core compendium containing all 534 expression profiles, keeping only the 3,886 core genes. The analysis of the strain data sets included all genes for the individual strain.

### Compiling the Salmonella Typhimurium RNA-seq compendium.

To compile the RNA-seq compendium, we searched the NCBI Sequence Read Archive (SRA) (https://www.ncbi.nlm.nih.gov/sra) for all publicly available Salmonella enterica RNA-seq data as of 20 August 2020. The strains and serovars were labeled using SRA metadata, when available, or linked literature (see Fig. S1 at https://github.com/AnnieYuan21/modulome-Salmonella/blob/main/Figures/Supplements/Additional/Fig_S1.pdf). In total, the initial compendium contained 1,444 expression profiles across 17 serovars. Within serovar Typhimurium, there were 1,174 expression profiles across eight strains.

Each expression profile was processed using a standardized pipeline (17). After low-quality samples were discarded, the final *S*. Typhimurium compendium contained 534 expression profiles from 46 BioProjects distributed across six *S*. Typhimurium strains (Fig. 1c).

### Independent component analysis captured the transcriptional regulatory network of S. enterica serovar Typhimurium.

To infer the TRN of Salmonella Typhimurium, we applied ICA to the core *S*. Typhimurium RNA-seq compendium, and this resulted in the identification of 115 iModulons. Together, these 115 iModulons captured 75% of the variance in gene expression (see Fig. S2). Since the iModulons represent underlying regulatory mechanisms, they can directly explain 75% of the variation resulting from transcriptional regulations. We note that this differed from the description of variation in data sets by the familiar principal-component analysis, which describes principal directions of variations in the data but is, unlike ICA, silent on knowledge-based explanations (18).

10.1128/msystems.00467-22.2 FIG S2 Explained variance plot for all core iModulons. Bar plot showing the explained variance of all 115 core iModulons. The iModulons with the top 10 explained variance are presented in Fig. 1d. Download FIG S2, PDF file, 0.4 MB. Copyright © 2022 Yuan et al. 2022 Yuan et al. https://creativecommons.org/licenses/by/4.0/ This content is distributed under the terms of the Creative Commons Attribution 4.0 International license.

To characterize the 115 iModulons, we first constructed a draft TRN of *S*. Typhimurium using a comprehensive TRN for the closely related model species Escherichia coli (9). We subsequently compared each iModulon against each draft regulon and found that 60 of the 115 iModulons had significant overlap with known regulons. These 60 regulatory iModulons recapitulated the structure of known regulons and demonstrated that a TRN established for a related model species can be applied to characterize closely related species.

**FIG 2:**
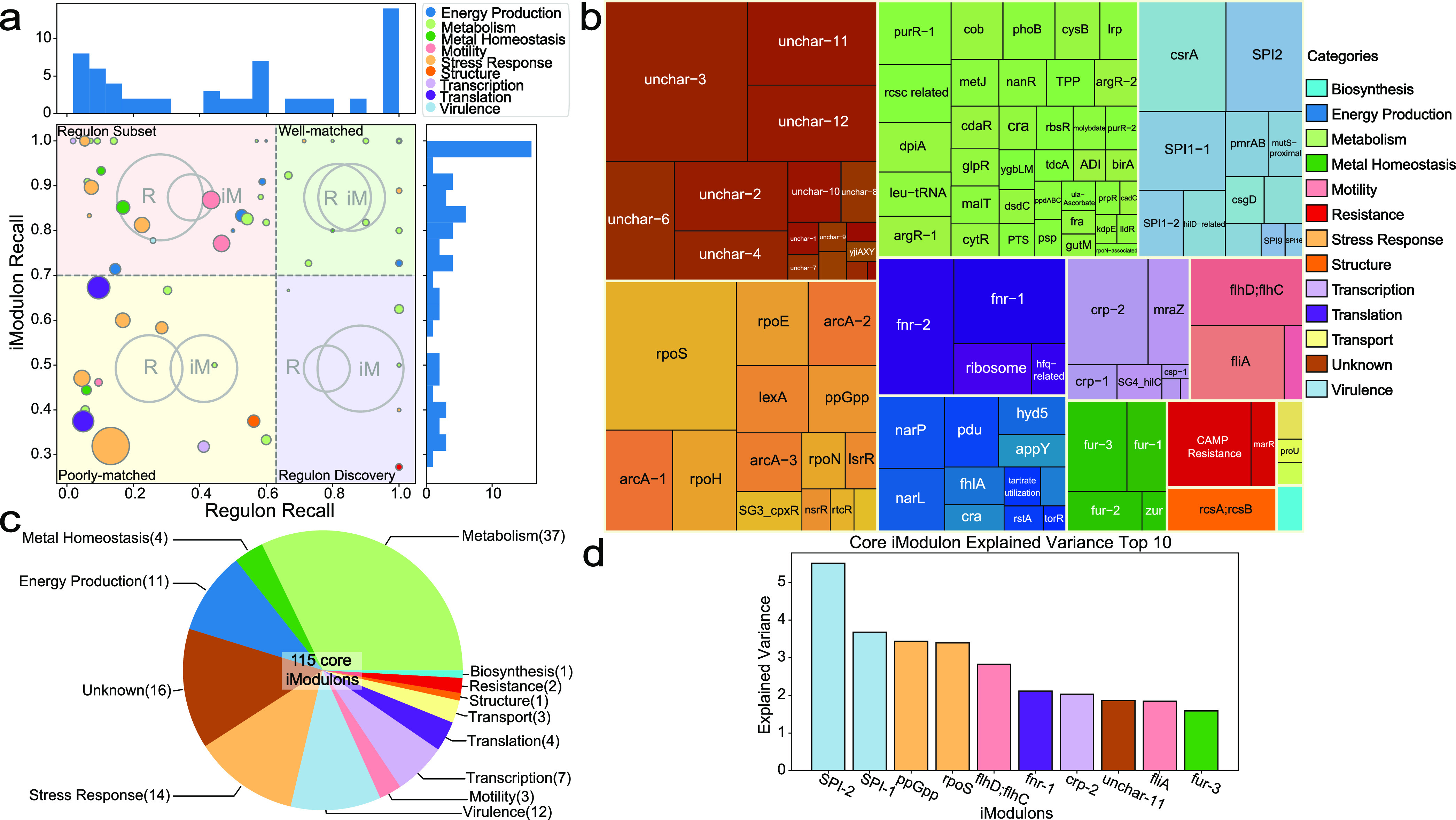
Core iModulon statistics. (a) Scatterplot of all the regulatory iModulons with histograms on the side. Each dot represents a regulatory iModulon. The size of the dot is determined by the size (i.e., number of genes) of the iModulon. Regulon recall was defined as follows: [(number of shared genes between iModulons and regulon)/(number of genes in a regulon)]. iModulon recall was defined as follows: [(number of shared genes between iModulons and regulon)/(number of genes in an iModulon)]. High iModulon recall and regulon recall values indicate high consistency of the iModulon with a previously characterized regulon. The regulatory iModulons are divided into four quadrants: regulon subset (top left), well-matched (top right), poorly matched (bottom left), and regulon discovery (bottom right). The relationship between the regulon (R) and iModulon (iM) in each quadrant is shown by the Venn diagram in the background. (b) Core iModulons and their functional categories. The functional categories of 115 core iModulons are presented in this tree map. The box sizes represent the sizes of the iModulons. A tree map with the names of all iModulons can be found in Fig. S3 in the supplemental material. (c) The number of iModulons in each category, shown as a pie chart. (d) Ten iModulons with the highest explained variance. iModulons with important functions, such as virulence, stress response, global regulatory network, and motility, were captured.

The relationship between regulatory iModulons and known regulons can be measured using two metrics: iModulon recall, which is the fraction of iModulon genes that belong to the regulon, and regulon recall, which is the fraction of regulon genes that belong to the iModulon (Fig. 2a). Many regulatory iModulons exhibit high recall rates, demonstrating good agreement between the iModulon TRN and the draft TRN structure based on E. coli.

10.1128/msystems.00467-22.3 FIG S3 Core iModulon functional categories. The complete tree map with all the iModulon names and functional categories labeled. Download FIG S3, PDF file, 0.4 MB. Copyright © 2022 Yuan et al. 2022 Yuan et al. https://creativecommons.org/licenses/by/4.0/ This content is distributed under the terms of the Creative Commons Attribution 4.0 International license.

The remaining 55 iModulons were characterized by information from public databases and literature (see Text S1 [Note 2]). We defined functional iModulons as iModulons that contained genes enriched for a specific function but were not linked to a specific transcriptional regulator (9). In addition, genomic iModulons were defined as iModulons that accounted for changes to the genome, such as single-gene knockouts or large deletions of genomic regions. To further evaluate the scope of the iModulon structure, we assigned each iModulon to a functional category (Fig. 2b and c). The two iModulons that captured the highest fraction of expression variance were functional iModulons related to the Salmonella pathogenicity islands (SPI-2 and SPI-1). Other iModulons that explained large fractions of expression variance included stress response iModulons (ppGpp and RpoS) and motility iModulons (flhD and flhC), indicating the importance of these mechanisms (Fig. 2d). Uncharacterized iModulons with high explained variances (e.g., unchar-11) could represent important biological functions and thus are possible targets for further studies.

### The CRP iModulon elucidated the structure of the *S*. Typhimurium CRP network and its effect on antibiotic susceptibility.

**FIG 3:**
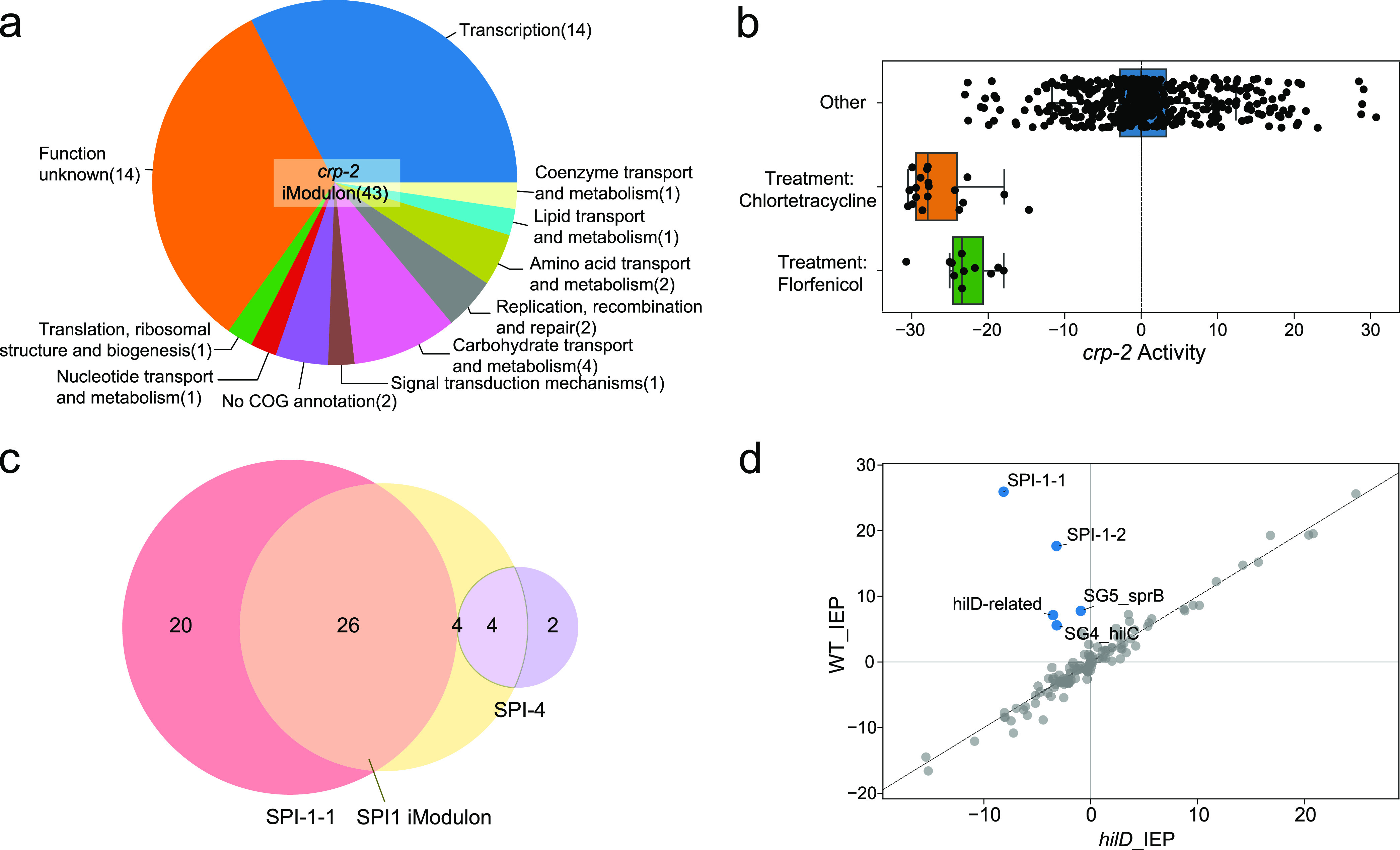
iModulons expanded the CRP target genes and revealed novel antibiotic responses, and they show that SPI-1 activity levels are consistent with the known regulatory role of hilD. (a) Gene function category breakdown of the *crp* iModulon. The numbers in parentheses give the number of genes in each category. (b) The activity level of the crp iModulon under the treatments of chlortetracycline and florfenicol (PRJNA344670). The activity of this iModulon significantly decreased under both conditions. (c) Relationship between the SPI-1 and SPI-4 pathogenicity islands and the SPI-1-1 iModulon (the larger SPI-1 iModulon). The SPI-1-1 iModulon contains 26 genes located in SPI-1 and 4 genes found in SPI-4. (d) DIMA plot for wild-type samples and *hilD* knockout samples in intermediate exponential phase (IEP) (PRJNA315446). iModulons related to SPI-1, SPI-4, and the coordination between these two islands were found to have differential activity levels between wild-type and *hilD* knockout samples (PRJNA315446).

CRP is a global regulator that orchestrates a variety of biological pathways. In E. coli, CRP is known to control the expression of 70 transcription factors, affecting over 300 gene targets involved in metabolism and stress response (19). Not surprisingly, in *S*. Typhimurium, CRP also plays important roles, and deletion of *crp* significantly affects virulence and metabolism (20). Although the complete CRP network of Salmonella is yet to be defined, the *crp-2* iModulon provides an initial draft to its gene membership and offers guidance to confirm targets of CRP. This *crp-2* iModulon contains 43 genes, 15 of which are known to be regulated by CRP (21). Fourteen genes in the iModulon are transcription factors that subsequently regulate additional biological activities (Fig. 3a). There are also 14 uncharacterized genes in the iModulon that make good targets for further studies.

The activity levels of each iModulon under different conditions indicate the activity of the underlying biological signal and regulator. The activity of the *crp-2* iModulon showed that it is strongly repressed under two conditions with antibiotic treatments (Fig. 3b). The activity of the iModulon drastically dropped when multidrug-resistant (MDR) samples were treated with chlortetracycline and florfenicol, antibiotics that are frequently administered to prevent diseases. It is known that the deletion of *crp* increases Salmonella’s resistance to fluoroquinolone antibiotics, possibly by affecting the drug delivery system and altering the drug target with DNA supercoiling (22), but the change in expression of the *crp* regulon under the treatment of tetracyclines or florfenicol is undocumented. Unlike fluoroquinolone, which inhibits DNA topoisomerases, both chlortetracycline and florfenicol work by interacting with the bacterial ribosome to prevent peptide synthesis. It is possible that downregulation of the *crp-2* iModulon in MDR samples results from the regulation of drug deliveries, similar to the mechanism to resist fluoroquinolone. An alternative hypothesis is that among the targets of *crp*, some ribosomal protein genes or other translation-related genes can influence the antibiotic’s interactions with the ribosome. Thus, the repression of the *crp* regulon could be part of the response mechanism of the MDR strains to reduce their susceptibility to antibiotics.

### iModulons identify regulators of the Salmonella pathogenicity islands.

Many of the virulence genes of Salmonella are located in Salmonella pathogenicity islands (SPIs) (23). SPIs contain important genes for invasion, survival, and infection. There are 12 common SPIs in *S*. Typhimurium (24). From the RNA-seq compendium that we compiled, ICA extracted five iModulons related to four SPIs (two SPI-1 iModulons and one iModulon each for SPI-2, SPI-9, and SPI-16) in the core genome. Together, the SPI-1-1 and SPI-2 iModulons explained nearly 10% of the expression changes in the compendium (Fig. 2d).

SPI-1 encodes a type three secretion system (T3SS) that delivers effector proteins to help Salmonella penetrate the intestinal epithelium (25). There are 46 genes located in SPI-1 (26), which includes genes coding for the secretion system apparatus, effector proteins, and their regulators. ICA extracted two iModulons (SPI-1-1 and SPI-1-2) enriched for SPI-1 genes, in total accounting for 5% of the global expression variance across the compendium. The largest SPI-1 iModulon (SPI-1-1) contains not only genes from SPI-1 but also four genes from SPI-4 (Fig. 3c). It is known that both SPI-1 and SPI-4 are required for Salmonella’s entry into polarized epithelial cells (27), and studies have shown that transcriptional factors encoded in SPI-1, such as *hilA*, *hilC*, *hilD*, and *sprB*, have regulatory effects on SPI-4 genes (28, 29). *hilD* resides in SPI-1 and codes for the important virulence regulator HilD. HilD regulates many virulence genes in SPI-1 directly and interacts with other virulence-related regulators, including HilA, HilC, and RtsA (outside of SPI-1). HilD forms a feed-forward loop with HilC and RtsA to amplify the regulation on HilA, which is known to regulate sprB and genes in SPI-4 (28, 30). Consistent with prior studies, iModulons related to *hilC*, *hilD*, and *sprB* have lower activities along with the two SPI-1 iModulons in the *hilD* mutant samples (PRJNA315446) (Fig. 3d). However, *hilC*, *hilA*, *sprB*, and *rtsA* mutants did not affect the activity of SPI-1 iModulons compared to the wild type. This confirmed the significance of HilD in the regulation of SPI-1 and provided additional support to previous findings that HilD has a more dominant role than HilC and RtsA (30, 31).

### Gene composition of pathogenicity island iModulons differed across the pan-genome.

**FIG 4:**
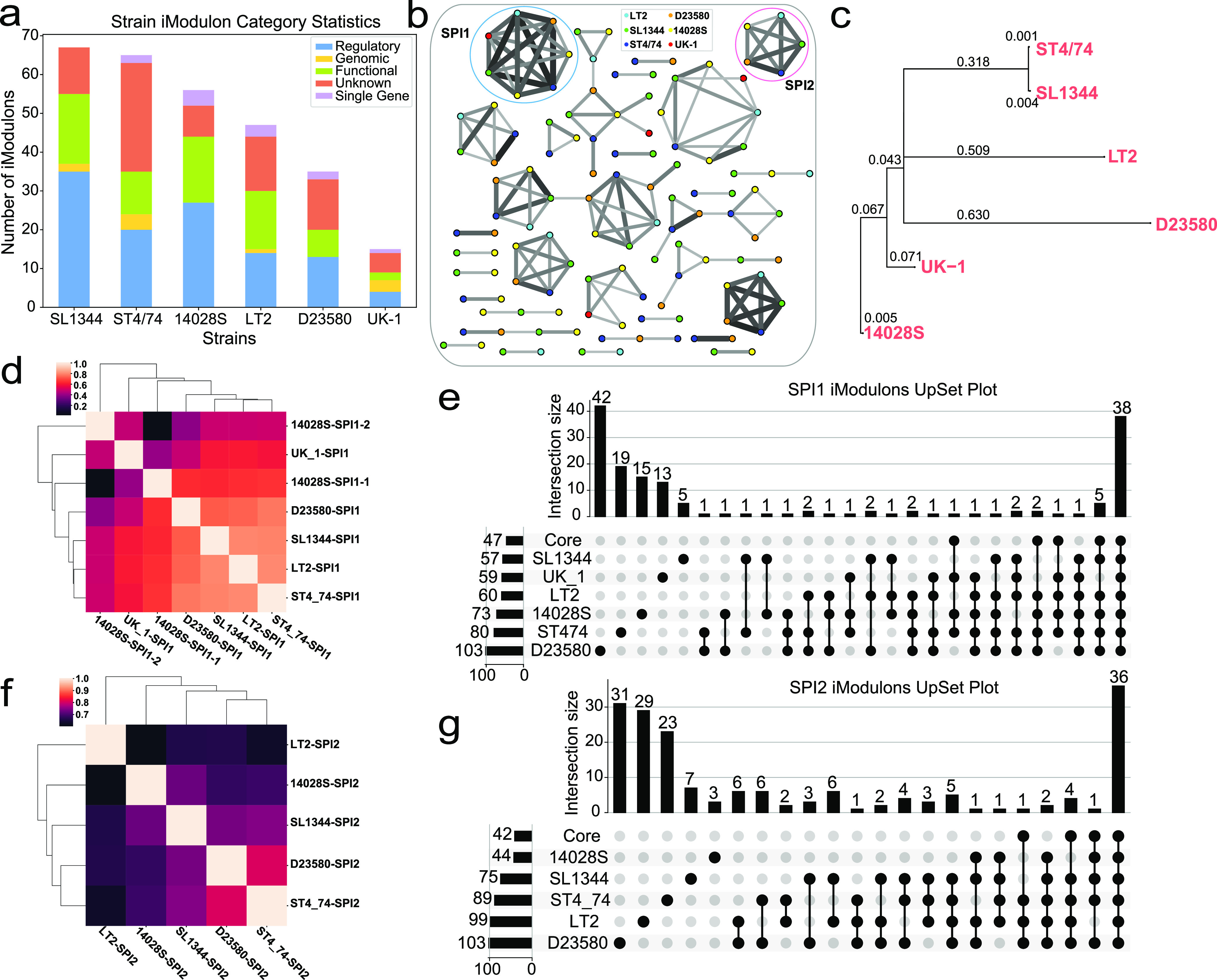
iModulon comparisons in different strains. (a) The number and category of iModulons for all six strains. (b) Reciprocal best hit (RBH) graph of iModulons from six strain data sets. Each node is an iModulon, and each edge is the RBH. The color of the node corresponds to the strains and the thickness of the edge indicates gene weighting similarity. The cutoff chosen in generating this RBH graph was a Pearson *R* value of 0.3 (58). The two clusters that are circled indicate the SPI-1 and SPI-2 iModulons. The full labeled RBH graph can be found in Fig. S4. (c) Simplified phylogenetic tree with only the six strains used for iModulon analysis. The branch lengths are indicated by the scores. (d) Cluster map for all SPI-1 iModulons in the six strains. (e) Upset plot to compare the gene contents of the SPI-1 iModulons across the core genome and the strains. The genes can be found in Table S1. (f) Cluster map for all SPI-2 iModulons in the five strains (UK-1 does not have a SPI-2 iModulon). (g) Upset plot to compare the gene contents of the SPI-2 iModulons across the core genome and five strains. The genes can be found in Table S2.

iModulons from the core genome can inform us about the TRN of serovar Typhimurium. However, different strains within the serovar have different properties, and these properties can be investigated using strain-specific iModulon gene composition. Here, we examined how evolutionary pressures shape both the genome composition and the TRNs of individual strains. We generated six strain-specific RNA-seq data sets, performed ICA on them, and obtained six sets of strain iModulons. The number of iModulons and their category breakdown are presented in Fig. 4a. With both the core and all the strain iModulons, we were able to do some comparisons.

10.1128/msystems.00467-22.4 FIG S4 Complete RBH graph. This figure is the fully labeled version of Fig. 6b (cutoff at a Pearson *R* value of 0.3). Download FIG S4, PDF file, 0.3 MB. Copyright © 2022 Yuan et al. 2022 Yuan et al. https://creativecommons.org/licenses/by/4.0/ This content is distributed under the terms of the Creative Commons Attribution 4.0 International license.

10.1128/msystems.00467-22.7 TABLE S1 The gene contents of the SPI-1 iModulons across the core genome and the six strains. Download Table S1, CSV file, 0.01 MB. Copyright © 2022 Yuan et al. 2022 Yuan et al. https://creativecommons.org/licenses/by/4.0/ This content is distributed under the terms of the Creative Commons Attribution 4.0 International license.

10.1128/msystems.00467-22.8 TABLE S2 The gene contents of the SPI-2 iModulons across the core genome and five strains. Download Table S2, CSV file, 0.01 MB. Copyright © 2022 Yuan et al. 2022 Yuan et al. https://creativecommons.org/licenses/by/4.0/ This content is distributed under the terms of the Creative Commons Attribution 4.0 International license.

If iModulons with similar composition and function are present in more than one strain, investigating the differences in iModulon composition for each strain can help us understand strain-specific genetic signatures that account for differences in phenotypic features. The reciprocal best-hit (RBH) graph in Fig. 4b shows clusters of similar iModulons in the strains. Here, we used the SPI iModulons as an example. Four SPIs were identified in the core genome, with SPI-1 and SPI-2 having the highest explained variance. Both of these two islands are very important for the virulence of *S*. Typhimurium and showed up in the strain iModulons as well. SPI-1 was found in all six of the strains, while SPI-2 was found in five. Note that strain UK-1 does not have an SPI-2 iModulon. Since UK-1 does have an SPI-2 island, this observation could simply be the result of UK-1’s relatively small RNA-seq data set.

To help understand the iModulon differences, the phylogeny of the six strains are presented in a simplified phylogenetic tree in Fig. 4c with the phylogenetic distances labeled on the branches. The clustermap of all the SPI-1 iModulons in Fig. 4d shows that the SPI-1 iModulons across all the strains were overall correlated. Interestingly, strains such as LT2 (avirulent) and D23580 (ST313) were clustered with the two closely related ST19 strains ST4/74 and SL1344. Also, the two SPI-1 iModulons in strain 14028S had poor correlation with each other. The larger SPI-1 iModulon codes for the majority of the SPI-1 genes with various functions, including the regulators, invasion factors, and T3SS apparatus, while the smaller one codes mostly for effectors and invasion factors. While most genes unique to the 14028S SPI-1 iModulons are uncharacterized, one effector, GtgA, was identified that is specific to this strain.

The differences in the SPI-1 iModulon contents are shown by the upset plot in Fig. 4e. The strains and the core shared 38 genes in the SPI-1 iModulons, while many strains also had their unique gene sets related to the SPI-1 island. The most unique strain was D23580, with 42 unique genes in its SPI-1 iModulon. Strain D23580 is an extremely invasive ST313 strain associated with nontyphoidal gastroenteritis and systemic disease. A multiple-antibiotic-resistant regulatory gene, *marB*, was identified uniquely in the D23580 SPI-1 iModulon, which supported its multidrug-resistant phenotype. The sequence of D23580 demonstrated genomic degradation, a hallmark for host adaptation. Many pseudogenes in this strain are homologous to genes in typhoidal serovars, such as S. Typhi and *S.* Paratyphi, and so it is hypothesized to be an intermediate between nontyphoidal and typhoidal serovars (32). The unique genes in its SPI-1 iModulons were highly enriched for the KEGG flagellar assembly pathway (map02040) and the *flhDC* regulon. Flagellar motility is extremely important for the infection of epithelial cells. Flagellar genes are under tight control of regulators encoded in SPI-1, and they coordinate closely with the SPI-1 T3SS during infections (33, 34). The flagellar genes of D23580 have been previously shown to be differentially expressed compared to ST19 strain ST4/74. It was also found that D23580 has attenuated expression of flagellin, which allows it to cause minimal inflammatory response and reduce host cell death. This adaptation significantly enhanced its survival within macrophages and resembled Typhoidal serovars that also infect the host cells while causing minimal inflammatory responses (16, 32). The unique genes in the D23580 SPI-1 iModulon further strengthened the argument that flagellar genes play a crucial role in virulence and inflammation and account for the more invasive and resistant phenotype of D23580.

Compared to SPI-1, the SPI-2 iModulons were less correlated across the strains (Fig. 4f). Other than strains D23580 and ST4/74, the rest of the strains had low correlation scores for this iModulon. Nonetheless, all strains and the core still shared 36 genes in the SPI-2 iModulon (Fig. 4g). Strain D23580 again possessed the highest number of unique genes, five of which were part of the vancomycin resistance pathway (map02020). These genes point to the multidrug-resistant properties of this strain being tightly linked to virulence. Another interesting observation showed that the SPI-2 iModulon in strain LT2 contained six genes encoding an ATP-binding cassette (ABC) transporter, many of which belong to the *malEGFK* and the *proVXW* operons. These LT2-specific SPI-2-related genes are also known to be regulated by *rpoD* (10 genes) and *rpoS* (3 genes). A defective *rpoS* gene is found to be the sole cause of avirulence of LT2 (35). The rest of the virulence mechanisms are assumed to be complete. It is unclear why these genes show a close relationship with the LT2 SPI-2, but investigations of these genes might lead to deeper understanding of the genomic roots of LT2’s unique phenotype.

iModularization of the transcriptomes of many strains of a subspecies can thus reveal differences in strain regulatory strategies that may be the basis for differences in pathogenicity and virulence.

### Three transport iModulons in the core genome were activated by antibiotic stress in MDR samples.

The emergence of MDR strains poses a serious challenge in preventative care and infection treatment. To help understand how these strains adapt to antimicrobial agents, we investigated three iModulons in the core genome (the *proU* iModulon, the molybdate iModulon, and the NikR iModulon) that had high activities under antibiotic treatment of MDR strains in PRJNA344670 (Fig. S5).

10.1128/msystems.00467-22.5 FIG S5 Transport iModulon activity under antibiotic treatments. The bar plots show the activities of three transport iModulons under the treatment of chlortetracycline and florfenicol. The color of the bar indicates the type of antibiotics, while the *x* axis shows the concentration. The numbers below the concentration are the specific MDR strains of the samples. (a) molybdate iModulon; (b) proU iModulon activity; (c) NikR iModulon activity. Download FIG S5, PDF file, 0.5 MB. Copyright © 2022 Yuan et al. 2022 Yuan et al. https://creativecommons.org/licenses/by/4.0/ This content is distributed under the terms of the Creative Commons Attribution 4.0 International license.

The *proU* iModulon (*proXWV*) encodes an osmoprotectant transport system activated by osmotic stress. When the MDR samples were treated with subinhibitory concentrations of antimicrobial agents, the activity of this iModulon drastically increased. It has not been documented that the *proXWV* operon is associated with other cellular functions in Salmonella, but iModulons indicate that the activation of this transporter can be triggered by antimicrobial agents.

The molybdate iModulon consists of seven genes (*modA*, *modB*, *modC*, STM0770, STM0771, STM3142, and *yghW*), many of which are a part of the essential molybdate transport system. While it is possible that more molybdenum is required for molybdoenzymes involved in the drug resistance mechanisms, the high activity for this iModulon in MDR samples implies that molybdate compounds affect bacterial survival under antibiotic treatments. Evidence has shown that silver molybdate and copper molybdate can enhance the abilities of antimicrobial agents in killing other Gram-negative bacteria, such as E. coli. The metal ions can disrupt the cell membranes, while the molybdenum oxide or molybdate oxyanion modulates the local pH to inhibit further growth (36). It is still unclear why *S*. Typhimurium MDR samples upregulate molybdate transport genes when treated with antibiotics, but this result strengthens the argument that molybdate has a major effect on the survival of Gram-negative bacteria, especially when antimicrobial agents are present.

Finally, the NikR iModulon consists of five putative ABC transporter genes in the NikR regulon (STM1255 to STM1259). Not only is this regulon important for the regulation of nickel uptake, but also all five genes are found in the quorum-sensing pathways as well (37). Previous studies have found that nickel chelator reduces the virulence and survival rate of multiple *Enterobacteriaceae*, including *S*. Typhimurium (38), by sequestration. The enhanced activity of the NikR iModulon under antibiotic treatment reinforced the hypothesis that nickel plays an important role in the survival of MDR samples and invited explorations for nickel-related methods to combat *S*. Typhimurium infections.

### Clustering of core genome iModulon activities illuminated antimicrobial resistance mechanisms.

**FIG 5:**
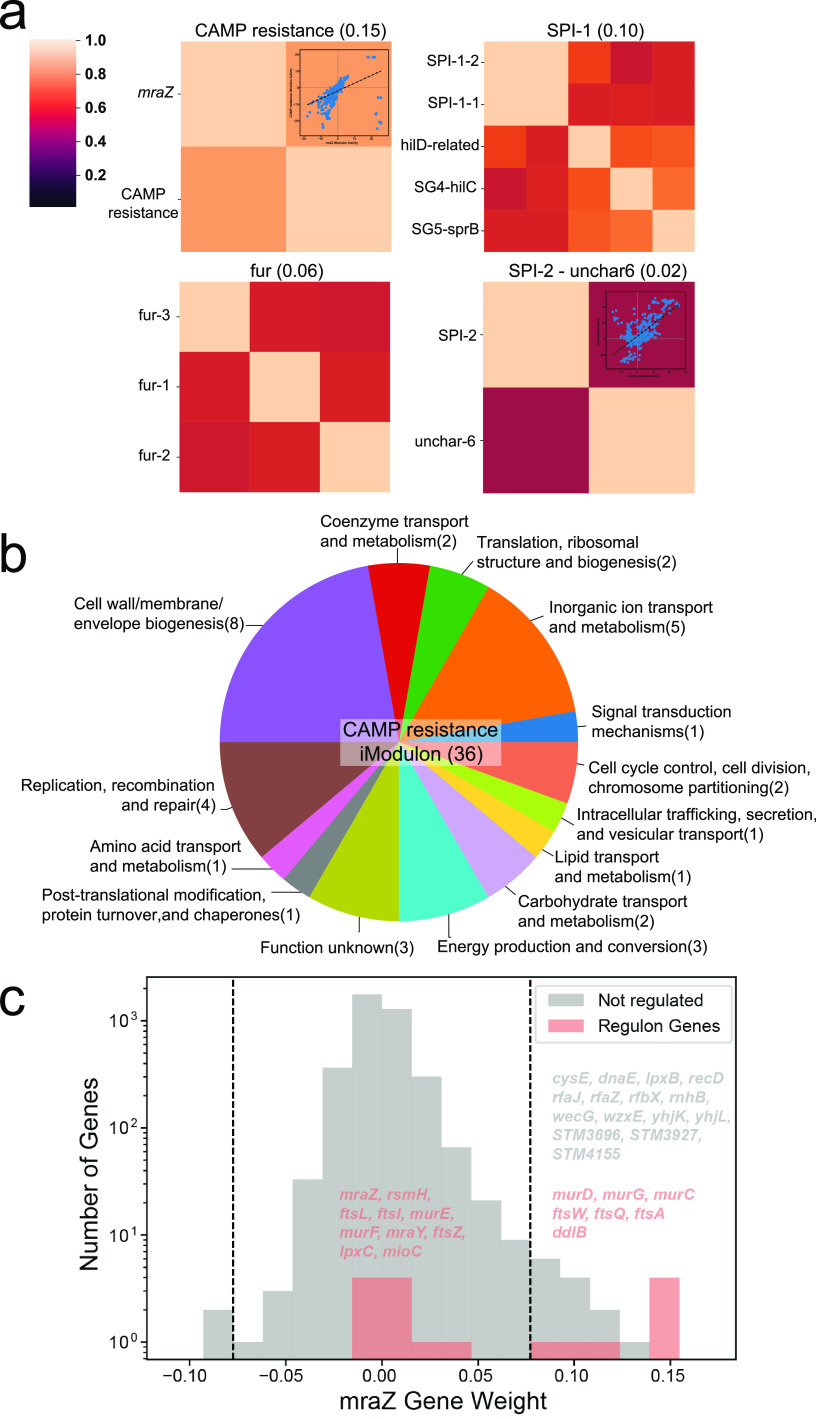
iModulon activity clusters show larger-scale coherency in transcriptomic responses. (a) Top four iModulon activity clusters with the highest correlations. (b) Gene function category breakdown of the CAMP resistance iModulon. This pie chart shows the number of genes in each functional category in the CAMP resistance iModulon. (c) Histogram of gene weights for the *mraZ* iModulon and regulon. The two dashed lines indicate the cutoff gene weight values for defining the iModulon. Genes in the *mraZ* regulon are indicated in red. Seven genes in the *mraZ* regulon were captured by the iModulon.

The activity fluctuations of individual iModulons under specific conditions help inform the functions of a single set of genes. However, biological processes often involve multiple gene sets with coordinated functions. These genes are not necessarily regulated by the same regulator, but they all respond together to the same stimulus, forming a “stimulon” (39). We identify stimulons by finding iModulons that had similar activities across the entire compendium (i.e., clustering iModulon activities). iModulons in the same cluster tended to have related biological functions, since their activities were correlated. For example, the cluster with a high correlation score contained five iModulons directly related to the SPI-1 pathogenicity island. The *fur* cluster consists of three iModulons related to the ferric uptake regulator Fur (Fig. 5a).

Given the clear relationships between iModulons in the SPI-1 and *fur* clusters, we used iModulon activity clustering to infer iModulon relationships and derive biological understanding. The cationic antimicrobial peptide (CAMP) resistance cluster was used to shed light on *S*. Typhimurium’s defense mechanism against CAMPs. Since the outer membrane is the first line of defense against CAMPs, Salmonella has developed ways to enhance resistance through modifying its surface. These defenses include reducing the negative charge on the surface to limit electrostatic interactions with CAMPs, regulating O-antigen length to create a stronger barrier, decreasing membrane fluidity to control CAMP intake, and increasing efflux of CAMPs (40).

In the CAMP resistance cluster, we captured two iModulons that potentially act in concert to resist antimicrobial peptides. The CAMP resistance iModulon contains 36 genes, 5 of which (*pqaB*, *sapD*, *sapF*, STM2300, STM2303) are in the CAMP resistance pathway from the KEGG Pathway database (stm01503). The genes in this iModulon have a variety of functions, including cell wall and membrane biosynthesis (Fig. 5b). The *mraZ* iModulon consists of 22 genes, 7 of which are regulated by *mraZ*, a gene within the *dcw* (division and cell wall) cluster, which has a major effect on cell division and peptidoglycan synthesis (Fig. 5c).

Many genes in the two iModulons are related to biosynthesis and transport of lipopolysaccharide (LPS). This result aligned well with the LPS modification hypothesis, where Salmonella needs to redesign its LPS to be less anionic to evade interactions with CAMPs. Genes related to O-antigen biosynthesis are present in both iModulons, pointing to the regulation of O-antigen length. We also found genes related to peptidoglycan (PG) synthesis in this iModulon cluster. There is little evidence of the direct involvement of PG remodeling in Salmonella’s defense response to CAMPs. However, PG is closely enveloped by LPS, so it is plausible that LPS remodeling works jointly with PG to protect the inner membrane from damage. Other than genes related to synthesis of the cell surface components, the presence of several transporters might be an indication of flux and binding regulation at the cell membrane. Moreover, we discovered several genes important for DNA replication, repair, and recombination. Antimicrobial agents such as CAMPs can induce DNA damage directly and indirectly, so these genes are likely part of the SOS response of the bacteria to repair and synthesize more DNA for survival. The rest of the genes with unknown functions are likely to be involved in the response to CAMPs, and this iModulon cluster offers insights into future investigations of antimicrobial resistance.

While SPI-1 is crucial for epithelial cell invasion, SPI-2 is required for intracellular replication, survival, and persistence. The SPI-2 iModulon explains 5.5% of the compendium-wide expression variance. It is clustered with an uncharacterized iModulon, uncharacterized-6 (Fig. 5a). The uncharacterized-6 iModulon contains many virulence and resistance-related genes, including *pagP*, *utgL*, the PhoP/Q two-component regulatory systems, and genes that this system regulates. These genes all show close relationships with the SPI-2 island. However, the relationships between virulence and many other genes are less obvious, and around half of the genes were characterized to code for putative proteins with unclear functions. These poorly characterized genes warrant further study to explore their relationship with pathogenicity islands and virulence.

### Uncharacterized iModulons provide a road map to discover future directions for investigation.

**FIG 6:**
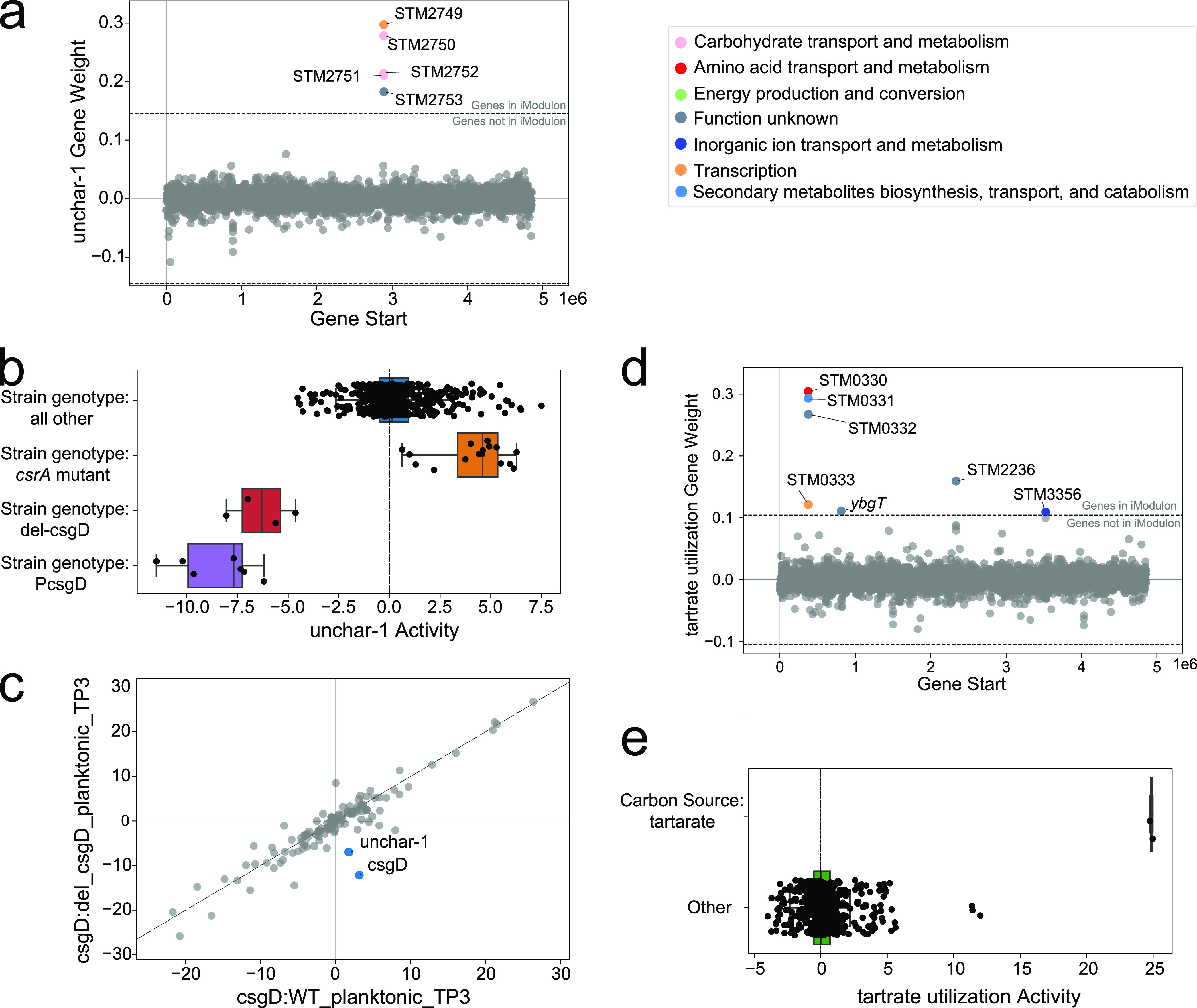
iModulons guide new discoveries. (a) iModulon composition and gene weights graph of the uncharacterized-1 iModulon. Five genes are in the iModulon, with one potential transcriptional regulator. (b) Activity of the uncharacterized-1 iModulon for *csrA* and *csgD* mutant samples. It can be seen that the iModulon had high activity when *csrA* was knocked out (KO) (PRJNA421560), or when *csgD* was disturbed (PRJNA280002) (59). (c) DIMA plot of a *csgD* KO sample and a wild-type sample in planktonic culture. (d) iModulon composition and gene weights graph of the tartrate utilization iModulon. (e) The activity of the tartrate utilization iModulon, where we saw increased iModulon activity when the samples were treated with the single carbon source tartrate (PRJEB4981) and when the strains carried a mutation in the Rho factor (PRJEB34015).

Many iModulons are consistent with existing knowledge, but others are not documented in the literature, thus providing directions for future research. In the core genome, one iModulon (uncharacterized-1) contains five genes related to the sorbitol transporter system (Fig. 6a). These five genes may compose a new operon, with STM2749 as the putative regulatory element. These genes are overexpressed in *csrA* mutant strains, suggesting that they are repressed by CsrA, a carbon storage regulator. Furthermore, the low activity of the iModulon in *csgD* mutant strains implies a regulatory role of *csgD* on these genes (Fig. 6b). In fact, only two iModulons have differential activity in the *csgD* mutant strain: the uncharacterized-1 iModulon and the CsgD iModulon (Fig. 6c). *csrA* regulates virulence, metabolism, and biofilm formation, while *csgD* is a central biofilm regulator in Salmonella. Therefore, we hypothesize that this gene cluster may be related to biofilm formation.

In another case, the tartrate utilization iModulon contains genes with a variety of different functions but that are not known to be regulated together (Fig. 6d). Interestingly, this iModulon had increased activity when tartrate was the sole carbon source (Fig. 6e) (see Text S1 [Note 3]). Among the seven genes in the iModulon, STM3356 is a putative cation transporter whose function is still unknown, but disruption of this gene can convert tartrate-fermenting phenotypes into tartrate-nonfermenting phenotypes for S. enterica serovar Paratyphi B dT+ (41). The presence of this gene in the tartrate utilization iModulon suggests that it is crucial for tartrate fermentation in *S*. Typhimurium as well. Although the remaining genes are poorly characterized, it is likely that they are related to tartrate utilization.

## DISCUSSION

Here, we constructed a pan-genome based on 506 sequenced genomes of representative *S*. Typhimurium strains. We then identified 400 iModulons across six strains based on available RNA-seq data. We established differential iModulon structures for the six strains, based on the pan-genome structure. Clustering of iModulon activities reflected the concept of simulons and revealed concerted gene regulations for complicated cellular responses. Furthermore, we showed that identified iModulons recapitulated the structure of known regulons and led to a series of new testable hypotheses. This study represents the first pan-genome-based analysis of strain-specific TRNs and has revealed insights into the genetic and regulatory bases for phenotypic signatures.

With pan-genomic analytics, we determined the phylogroup structure of serovar Typhimurium. We conducted iModulon analysis of six *S*. Typhimurium strains contained in our RNA-Seq compendium that represented the diversity in this serovar (Fig. 1a). Our iModulon results recovered well-studied regulatory features, including the CRP network, with high accuracy and discovered new potential regulatory targets of CRP. We also identified iModulons that reflected important Salmonella pathogenicity islands. Interestingly, while the six strains had many common regulatory structures, the coregulated gene sets of the SPI iModulons differed between strains. We observe unique flagellar and antibiotic resistance genes associated with the SPI1 iModulon of the multidrug-resistant strain D23580. These unique genes might govern D23580’s invasive and drug-resistant traits. We also found activations of prophage iModulons under various conditions that may reflect the phenotypic features in specific strains (Text S1 [Note 4]). These novel strain-specific results suggest that pan-genome-based iModulon analysis helps us to understand differential regulations across strains and links them to strain-specific phenotypic signatures. It can also provide fundamental insights into the evolutionary differences in regulator function between these six *S*. Typhimurium strains.

In *S*. Typhimurium’s core genome, we discovered three transport iModulons with increased activities in multidrug-resistant samples. We was also found that the *crp* iModulon had repressed activity under chlortetracycline and florfenicol treatment. Multidrug-resistant nontyphoidal Salmonella is becoming an increasingly serious threat to public health (42, 43). Therefore, these results open new avenues for investigating antibiotic resistance mechanisms to assist drug and treatment designs. The clustering of activities uncovered a group of iModulons that respond to the same stimuli, forming stimulons. We found an iModulon cluster potentially associated with the surface-remodeling process during CAMP resistance and other clusters for which we generated hypotheses about concerted biological processes. We identified three iModulons potentially involved in the bile shock response specific to the ST4/74 strain (Text S1 [Note 5] and Fig. S6). Finally, two iModulons with interesting activities will make good targets for regulon discovery related to biofilm formation and tartrate utilization.

10.1128/msystems.00467-22.6 FIG S6 Comparative studies revealed iModulons that responded to bile exposure in strain ST4/74. (a) Gene function category breakdown of the uncharacterized-12 iModulon. (b) Activity of the uncharacterized-5 iModulon across samples from all six strains. While the activity of this iModulon was close to zero for five strains, for strain ST4/74, several samples showed significantly decreased activity of this iModulon (these samples are from PRJNA215033, PRJNA315446, PRJNA393682, and PRJNA490148). No obvious patterns were seen in these samples with low activities. (c) Activity of the uncharacterized-12 iModulon across samples from all six strains. Samples from strains that showed significantly decreased activity were from PRJNA215033, PRJNA315446, PRJNA393682, and PRJNA490148). (d) DIMA plot for strain ST4/74 samples under bile shock against samples in LB at middle exponential phase. (e) Differential iModulon activity plot for samples under bile shock in strain ST4/74 and D23580. (f) iModulon activity clusters for three uncharacterized iModulons that showed low activity for samples under bile shock in strain ST4/74. Download FIG S6, PDF file, 2.6 MB. Copyright © 2022 Yuan et al. 2022 Yuan et al. https://creativecommons.org/licenses/by/4.0/ This content is distributed under the terms of the Creative Commons Attribution 4.0 International license.

iModulon analyses represent a new approach to understand TRNs and to provide biologically relevant hypotheses from large RNA-seq data sets (9–11, 18, 44). Unlike traditional differential gene expression analysis, which only compares two conditions, iModulons can simultaneously analyze hundreds of expression profiles and provide clustered interpretable results. Here, we have demonstrated that the combination of pan-genomics and iModulon analysis offers novel insights into the differential TRNs between closely related *S*. Typhimurium strains. This comparison produced key results linked to a regulatory basis for different virulence responses of different strains. As RNA-seq data are becoming less expensive to generate, we can look forward to detailed TRN elucidation of phylogroups and subclades of other pathogenic bacteria and a much deeper understanding of their differential regulatory characteristics. Demonstrated differences in virulence properties could lead to phylogroup-specific treatments which, in turn, would lead to improved antibiotic stewardship.

## MATERIALS AND METHODS

### Pan-genome analysis and phylogenetic tree.

We gathered the metadata of 3,329 Salmonella Typhimurium genomes from PATRIC (https://www.patricbrc.org/). To ensure genome quality, we removed genomes with less than 10× coverage and more than 150 contigs. Then, 500 genomes were selected randomly from all 1,199 *S*. Typhimurium genomes that passed quality control. We checked to include the strains for which we had RNA-Seq data, which resulted in a final 506 strains. Using CD-HIT (45) with a threshold of 0.9 (*n* value of 5 and *c* value of 0.9), we assembled the pan-genome matrix of these 506 strains. To identify the most “representative” strains, we used k-means clustering and identified the optimal number of clusters with both explained variance and silhouette scores. With a silhouette score of 0.127 and explained variance of 86%, 177 clusters were chosen, giving 177 centroid strains. These 177 strains were then used to construct the phylogenetic tree (generated with snippy and Gubbins [46] with default parameters, using the LT2 genome as a reference) and the *S*. Typhimurium core genome. Since some of the genomes of these strains were incomplete, we performed a sensitivity analysis to identify the size of the core genome. In the end, we used a cutoff of 4,209 clusters from 172 genomes (Fig. 1b). These clusters were defined in the PATRIC locus tags. To match with the locus tags of the expression profiles, PATRIC locus tags were mapped to the RefSeq LT2 locus tags. Some gene families were lost due to missing mapping between the two types of identifies, and the final core genome consisted of 3,886 genes with the RefSeq LT2 locus tag. The expressions of the core genome were extracted from the data sets of each strain and concatenated together to form the final core genome expression profile, with 3,886 genes and 534 samples.

### RNA-seq data acquisition, metadata curation, and preprocessing.

Following the PyModulon workflow (https://github.com/avsastry/modulome-workflow/tree/main/1_download_metadata), we compiled all of the RNA-seq data for Salmonella Typhimurium on NCBI SRA as of 20 August 2020. We performed manual curation of experimental metadata by inspecting literature associated with specific BioProject IDs documented in the metadata files. This was to identify different strains in the *S*. Typhimurium serovar and experimental conditions, such as number of biological replicates, culture media, growth phase, temperature, or any additional treatment that could assist us in understanding the results. Detailed metadata also helped with subsequent quality control steps. There were 1,049 samples with detailed metadata and in total 8 different *S*. Typhimurium strains in the data sets. Six strains had enough samples for subsequent analysis. Two strains (DT2 and sg_wt7) only had four samples each, and so they were marked in the phylogenetic tree in Fig. 1a and used to construct the core genome, but they were not investigated individually with ICA decomposition. The rest of the samples were then sorted into 6 separate files by strain and processed using the RNA-seq pipeline available at https://github.com/avsastry/modulome-workflow/tree/main/2_process_data. The final expression profiles were reported in units of log-transformed transcripts per million (log TPM).

### Quality control and data normalization.

The expression data sets were subjected to five quality control steps, as outlined at https://github.com/avsastry/modulome-workflow/tree/main/3_quality_control. Briefly, we checked four statistics from the FastQC report, which were the per-base sequence quality, per-sequence quality scores, per-base *n* content, and adapter content, and we discarded all samples that didn’t pass these four criteria. Then, we removed samples with less than 5 × 10^5^ reads mapped to coding sequences. After that, we clustered the samples using hierarchical clustering and removed samples that did not conform to a typical expression profile, as these samples often use nonstandard library preparation methods, such as ribosome sequencing and 3′- or 5′-end sequencing (47). Then, samples with poor replicate correlation (Pearson *R* score of <0.9) and with no biological replicates were discarded. However, there was an interesting project in which biofilm formation was observed over a time course (PRJNA280002), and so these samples were kept despite a lower Pearson *R* score (0.8) to study temporal activities of iModulons. Also, PRJNA490148 contains samples from comparative studies of two *S*. Typhimurium strains. We were interested to see the differences in iModulon activities for the two strains under the same conditions, and so we included samples from this project even though some of them did not have biological replicates. After quality control, 534 samples were kept for ICA. The metadata associated with these samples can be found in Table S3 and in our GitHub repository.

10.1128/msystems.00467-22.9 TABLE S3 Metadata file associated with all the samples. Download Table S3, CSV file, 0.3 MB. Copyright © 2022 Yuan et al. 2022 Yuan et al. https://creativecommons.org/licenses/by/4.0/ This content is distributed under the terms of the Creative Commons Attribution 4.0 International license.

To obviate any batch effects resulting from combining different expression profile data sets, we selected a reference condition in each project to normalize each data set. This ensured that nearly all independent components generated were due to biological variation rather than technical variation. This normalization allowed us to compare gene expression and iModulon activities within a project to a reference condition, but not across projects.

Six individual data sets were generated using the methods described above. To obtain the core genome expression profile, all gene locus tags were unified by using the LT2 locus tags from refseq, and then the expression profiles were combined together based on the core genome that we defined earlier. The final core genome expression profile consisted of 534 samples from 46 BioProjects distributed across 6 *S*. Typhimurium strains (Fig. 1c).

### Defining the optimal number of independent components.

To compute the optimal number of independent components, an extension of ICA was performed on the RNA-seq data set, as described by McConn et al. (48).

Briefly, the scikit-learn (v0.23.2) (49) implementation of FastICA (50) was executed 100 times with random seeds and a convergence tolerance of 10^−5^. The resulting independent components (ICs) were clustered using DBSCAN (51) to identify robust ICs, using an epsilon of 0.1 and minimum cluster seed size of 50. To account for identical with opposite signs, the following distance metric was used for computing the distance matrix: *d_x,y_* = 1 − ‖ρ*_x,y_*‖, where *ρ_x_*_,_*_y_* is the Pearson correlation between components *x* and *y*. The final robust ICs were defined as the centroids of the cluster.

Since the number of dimensions selected in ICA can alter the results, we applied the above procedure to each expression profile multiple times, ranging the number of dimensions from 5 to 380. Depending on the number of dimensionalities being tested, the step size varied from 5 (for smaller data sets with fewer dimensions) to 10 or 20 (for large data sets). The upper limit of the choice of dimensionality was approximately the number of samples in the data set.

To identify the optimal dimensionality, we compared the number of ICs with single genes to the number of ICs that were correlated (Pearson *R* > 0.7) with the ICs in the largest dimension (called “final components”). We selected the number of dimensions where the number of non-single-gene ICs was equal to the number of final components in that dimension (see Fig. S3 at https://github.com/AnnieYuan21/modulome-Salmonella/blob/main/Figures/Supplements/Additional/Fig_S3.pdf). The optimal number of dimensions for the core genome was 220. The optimal dimensionalities of the strains are presented in Fig. S4 at https://github.com/AnnieYuan21/modulome-Salmonella/blob/main/Figures/Supplements/Additional/Fig_S4.pdf.

ICA produces two matrices. The M matrix contains the robust independent components, and the A matrix contains the corresponding activities. The product of the M and A matrices approximates the expression matrix (the X matrix), which is the curated RNA-seq compendium. Each independent component in the M matrix is filtered to find the genes with the largest absolute weightings, which ultimately generates gene sets that make up iModulons. Implementing this process on the *S*. Typhimurium core genome resulted in 115 iModulons that explained 75% of the expression variance in the core compendium (Fig. S2). The number of iModulons from the strain expression profiles can be found in Fig. 4a.

### Compiling TRN and gene annotations.

The TRN file was generated using a predefined TRN for the closely related bacteria Escherichia coli (9) and the bidirectional blast results of *S*. Typhimurium LT2 and E. coli genomes. The mapping of locus tags of each studied strain to the STM locus tags of strain LT2 can be found in our GitHub repository (https://github.com/AnnieYuan21/modulome-Salmonella) in the pan-genome section under the folder bbh_results. The genes were annotated following the annotation pipeline that can be found at https://github.com/SBRG/pymodulon/blob/master/docs/tutorials/creating_the_gene_table.ipynb. Additionally, KEGG (52) and Cluster of Orthologous Groups (COG) information was obtained using EggNOG mapper (53). Uniprot IDs were obtained using the Uniprot ID mapper (54), and operon information was obtained from Biocyc (55). Gene ontology (GO) annotations were obtained from AmiGO2 (56).

### Computing iModulon enrichments.

iModulon enrichments against known regulons were computed using a two-sided Fisher’s exact test, with the false-discovery rate (FDR) controlled at 10^−5^ using the Benjamini-Hochberg correction. Functional enrichment through KEGG and GO annotations was similarly computed but with an FDR of <0.01. This pipeline can be found at https://github.com/SBRG/pymodulon/blob/master/docs/tutorials/gene_enrichment_analysis.ipynb. The genes in each individual iModulon can be found by visiting our website at https://imodulondb.org. They can also be found using the iModulon json files included in the GitHub repository, https://github.com/AnnieYuan21/modulome-Salmonella.

### Differential iModulon activity analysis.

The differences in iModulon activities under relevant conditions were calculated using a log-normal probability distribution. For each comparison, the absolute differences in the mean iModulon activities were calculated and compared to an iModulon log-normal distribution (calculated between biological replicates). *P* value statistics were obtained for each condition comparison across all iModulons, and an FDR score was calculated. iModulons with a difference greater than 5 and FDR less than 0.01 were considered significant. Differential iModulon activity plot (DIMA plots) can be generated to visualize iModulon activity differences under one condition against another.

### Calculating iModulon activity clusters.

The activities of iModulons were clustered using the Seaborn (57) clustermap function in Python. The default distance metric was the following: *d_x,y_* = 1 − ‖ρ*_x,y_*‖, where ‖ρ*_x,y_*‖ is the absolute value of the Spearman *R* correlation between two iModulon activity profiles. Other available distance metric options include Pearson R, Kendall rank, and mutual information. The option used for this project was mutual information. The threshold for optimal clustering was determined by testing different distance thresholds to locate the maximum silhouette score.

### Generating iModulonDB dashboards.

iModulonDB dashboards were generated using the PyModulon package (13, 17). The pipeline can also be found at https://pymodulon.readthedocs.io/en/latest/tutorials/creating_an_imodulondb_dashboard.html.

### Data availability.

The complete code to reproduce this pipeline can be found at https://github.com/AnnieYuan21/modulome-Salmonella. The ICA part of the workflow is adapted from Sastry et al. (17), which is also available at https://github.com/avsastry/modulome-workflow.
