# Supplementary figures and images for: Pan-Genome Analysis of Transcriptional Regulation in Six Salmonella enterica Serovar Typhimurium Strains Reveals Their Different Regulatory Structures
Source: mSystems. 2022 Nov 1;7(6):e00467-22. doi: 10.1128/msystems.00467-22 (PMC9764980; doi:10.1128/msystems.00467-22)

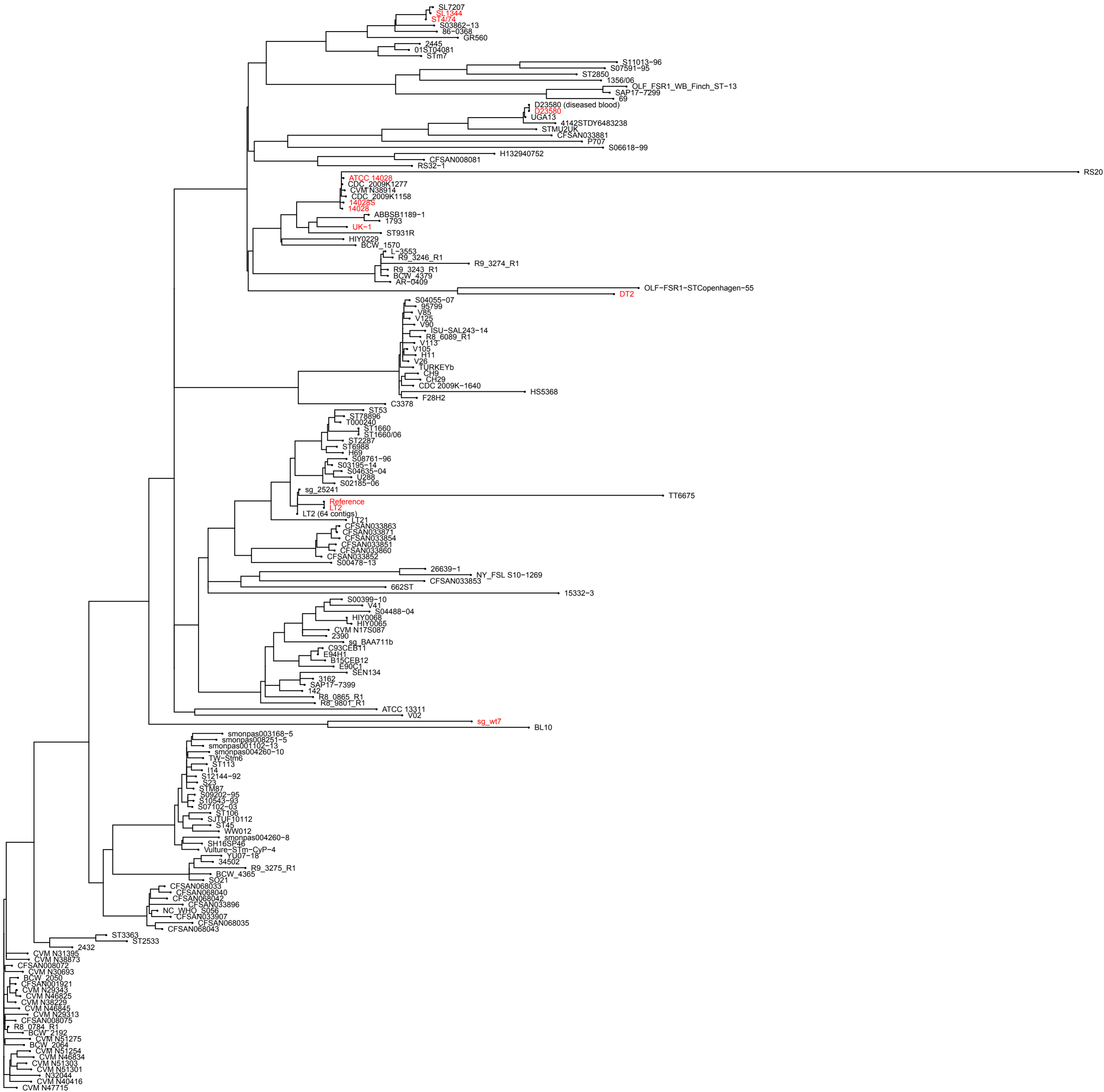

Supplement: FIG S1 [file msystems.00467-22-s0003.pdf]

Core iModulon Explained Variance

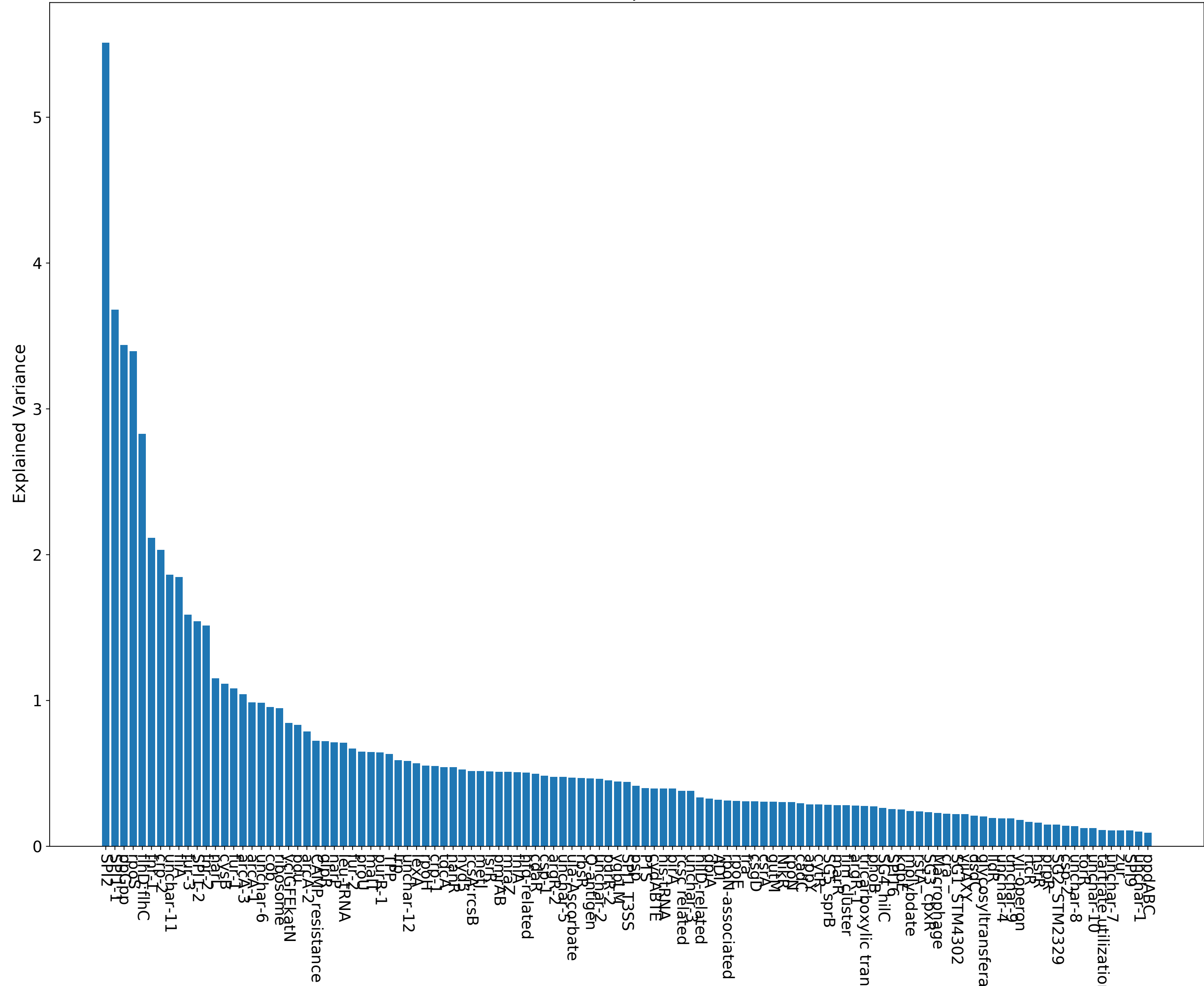

Supplement: FIG S2 [file msystems.00467-22-s0004.pdf]

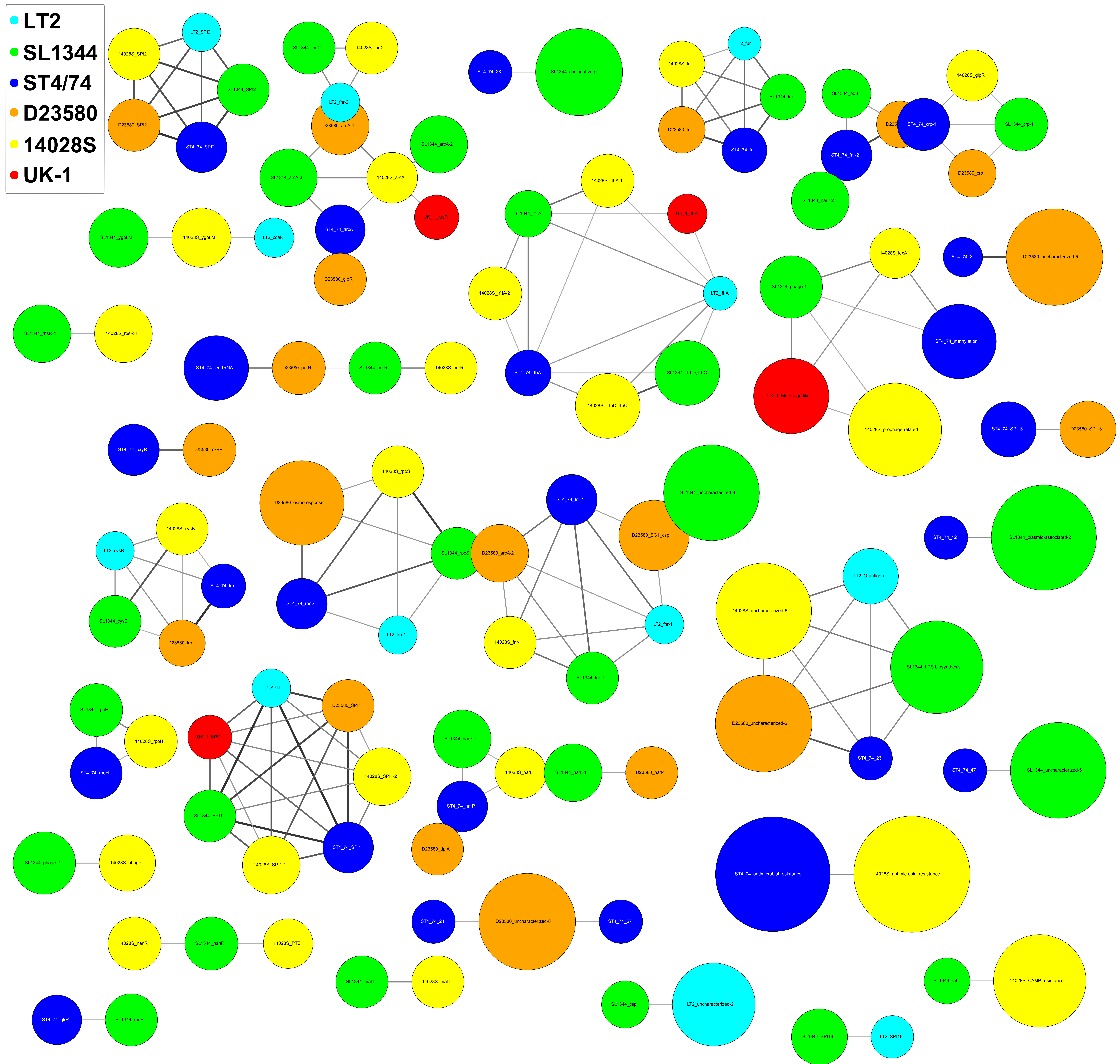

Supplement: FIG S4 [file msystems.00467-22-s0006.pdf]

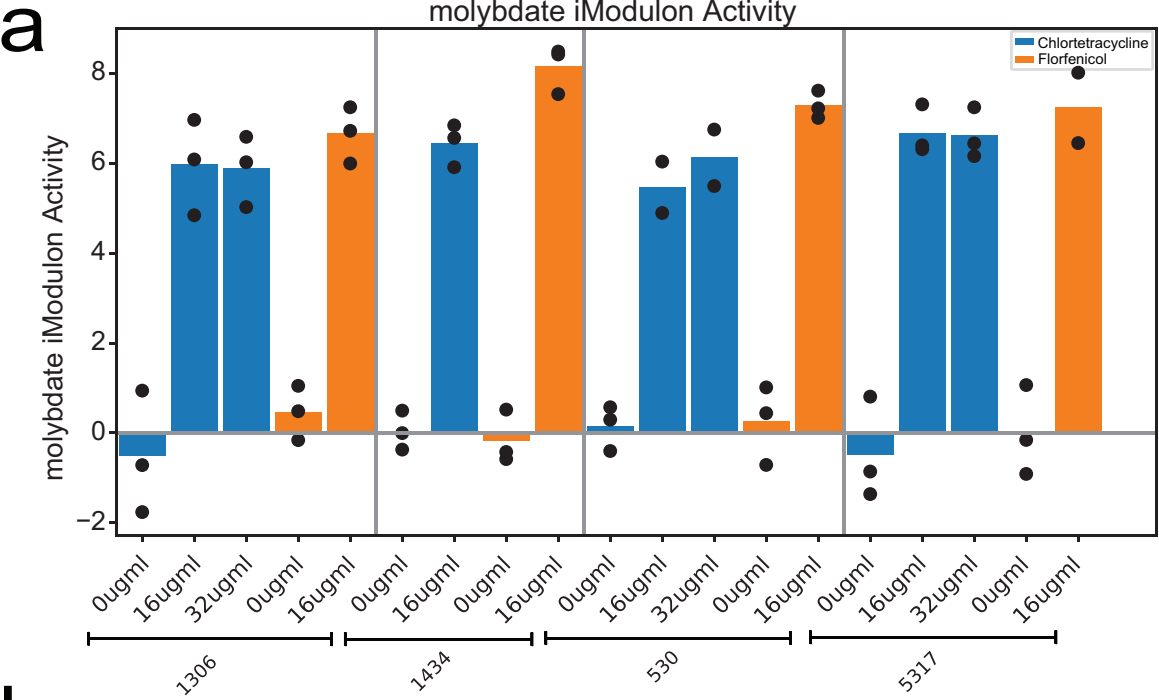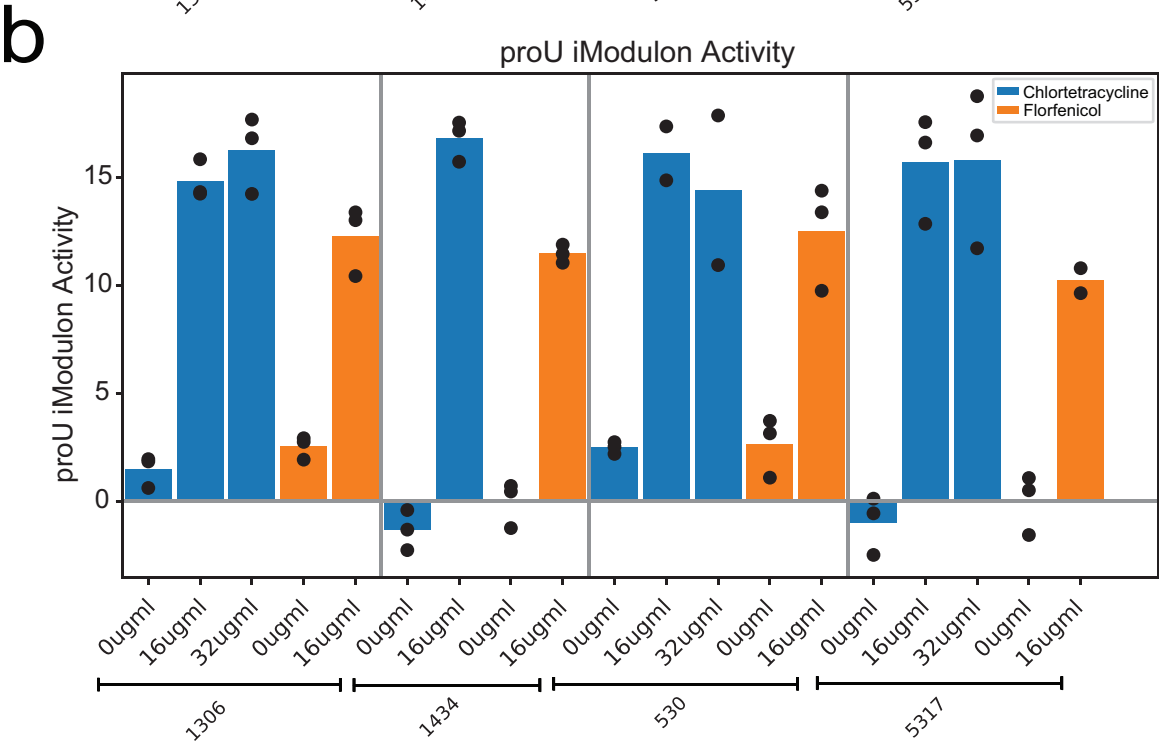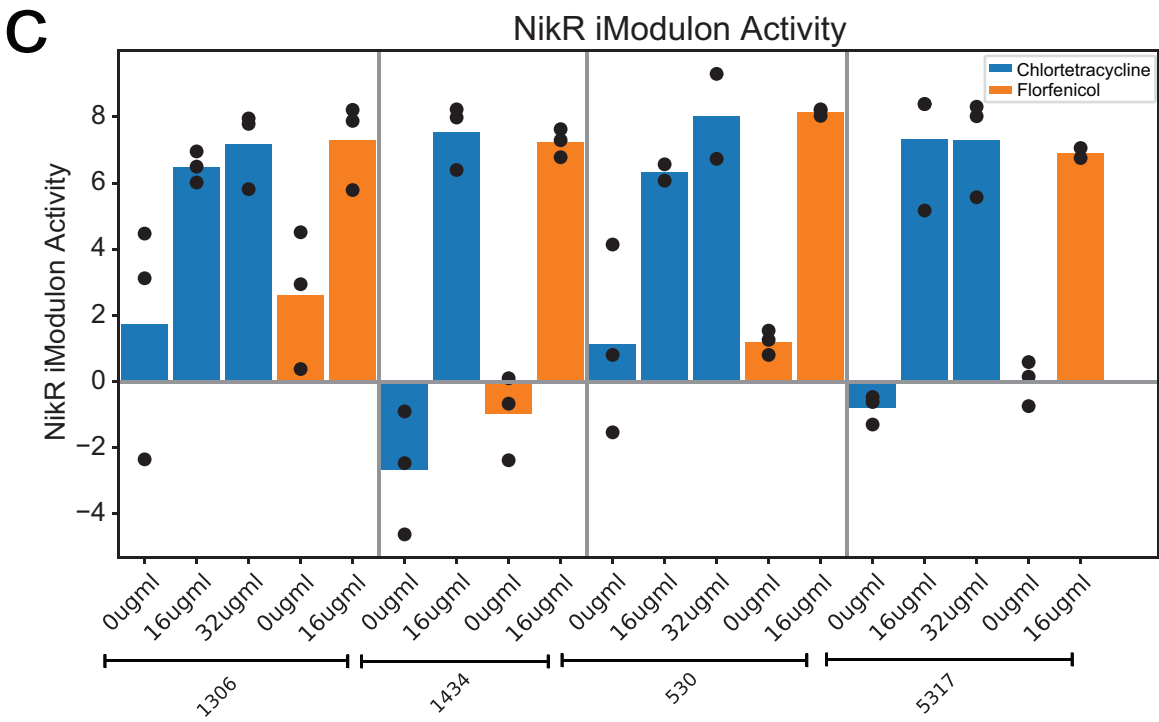

Supplement: FIG S5 [file msystems.00467-22-s0007.pdf]

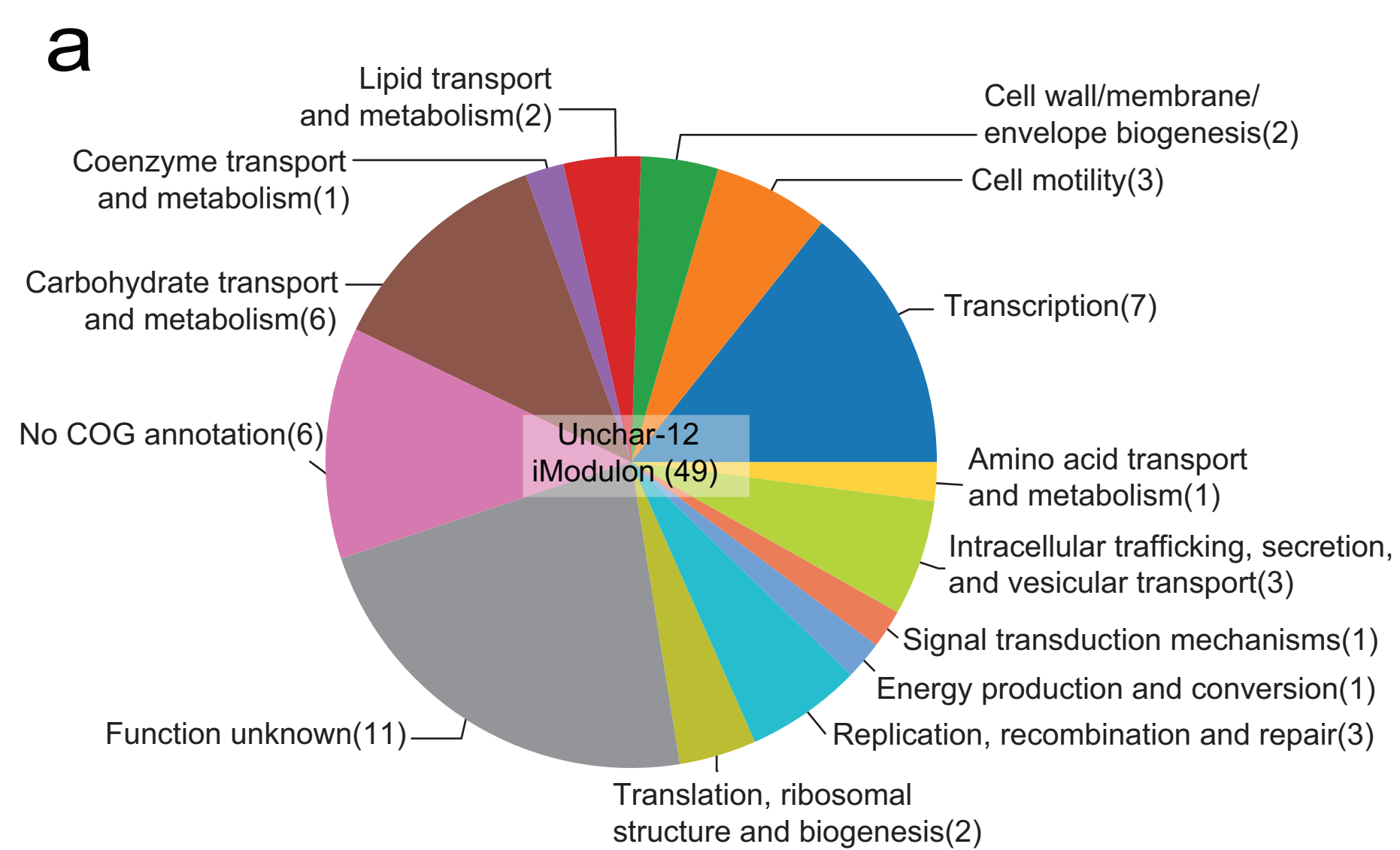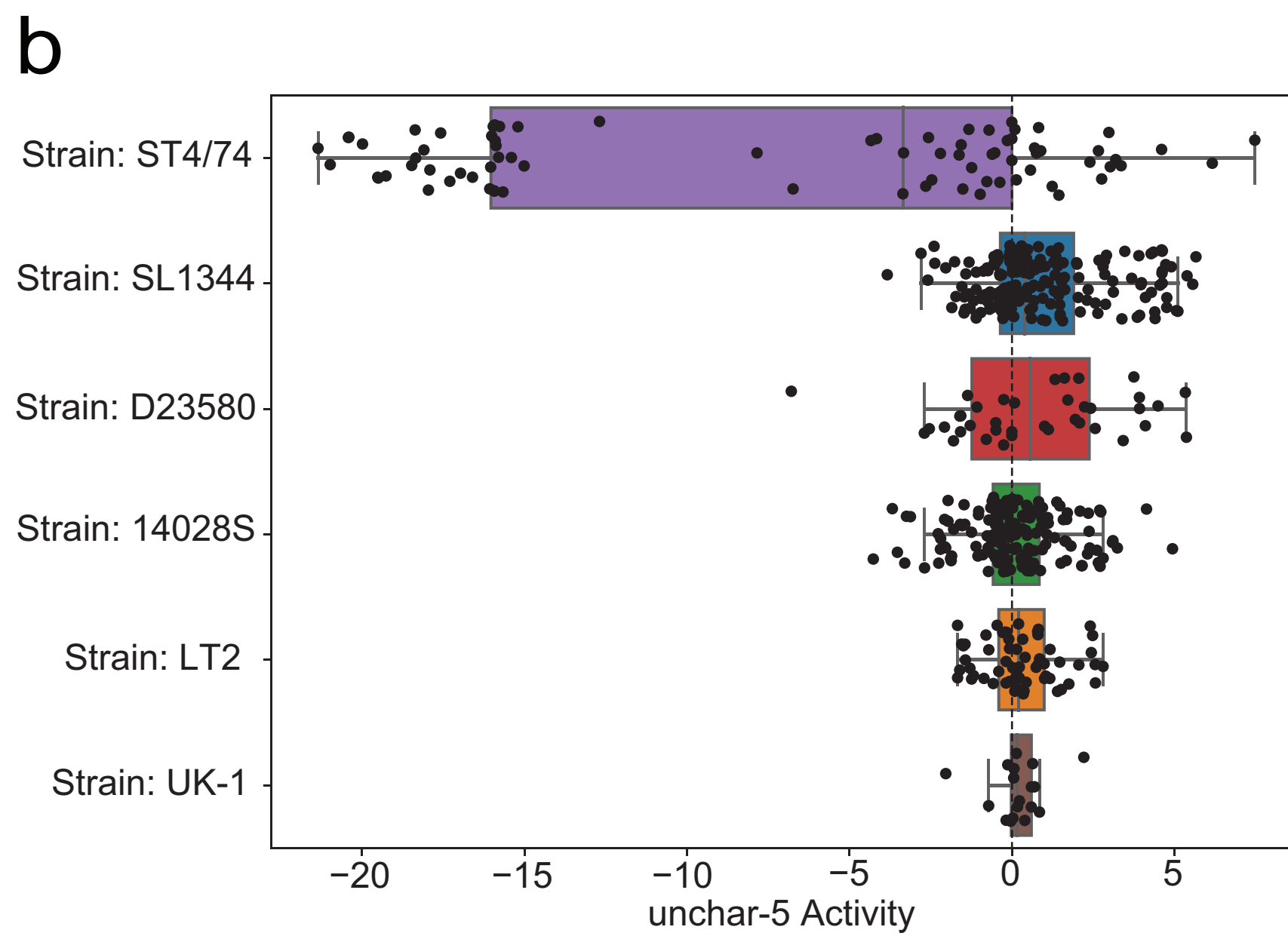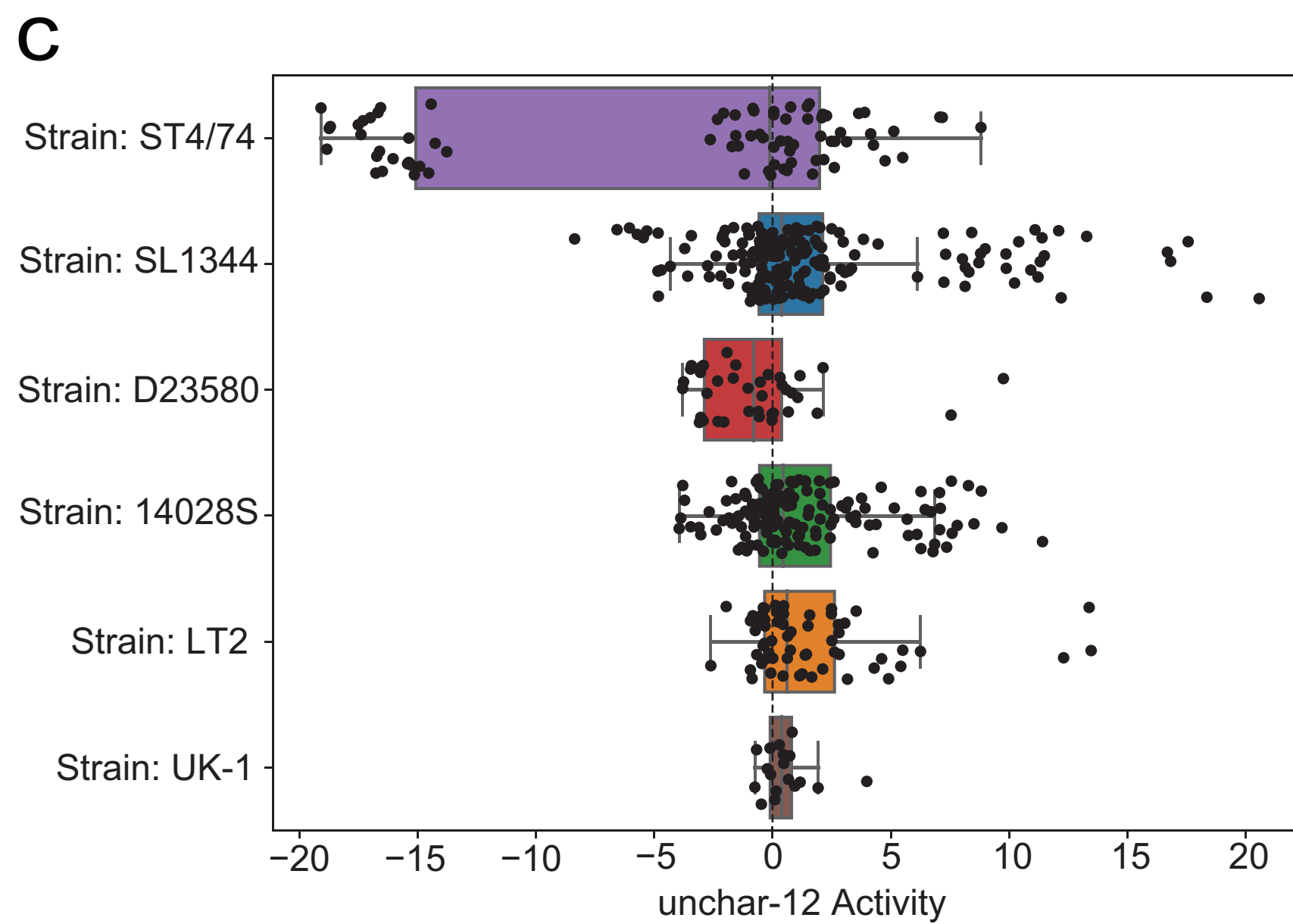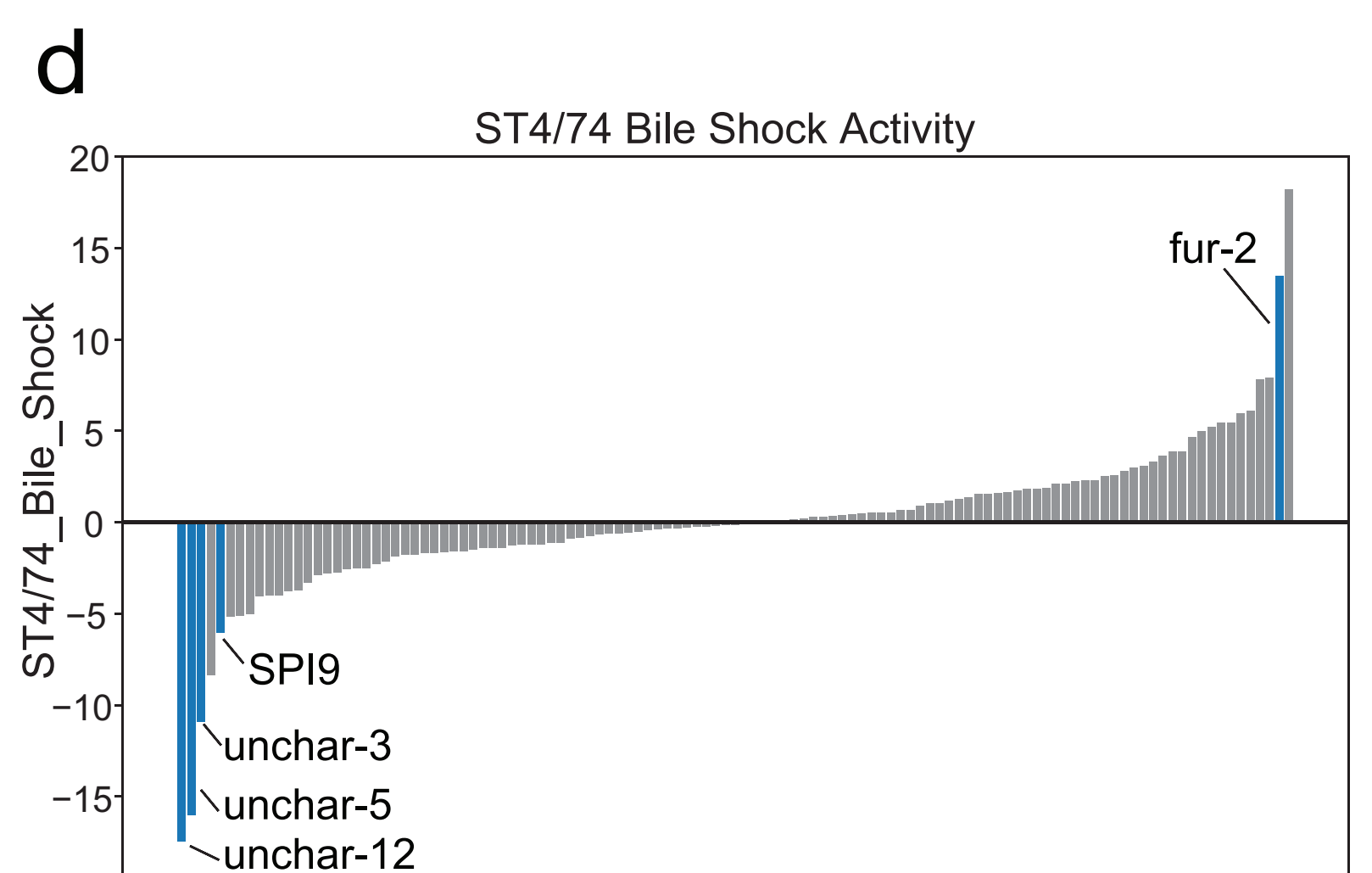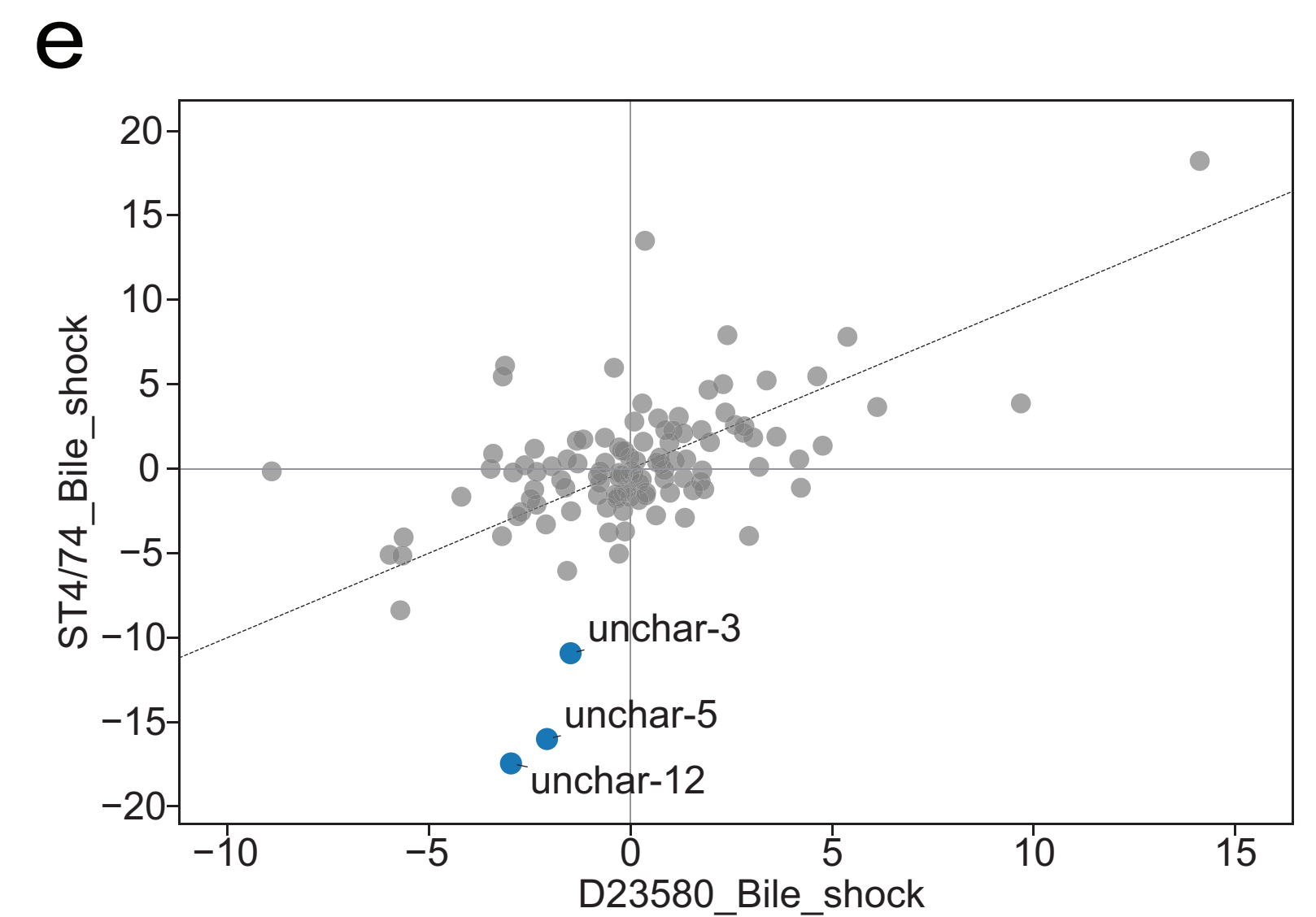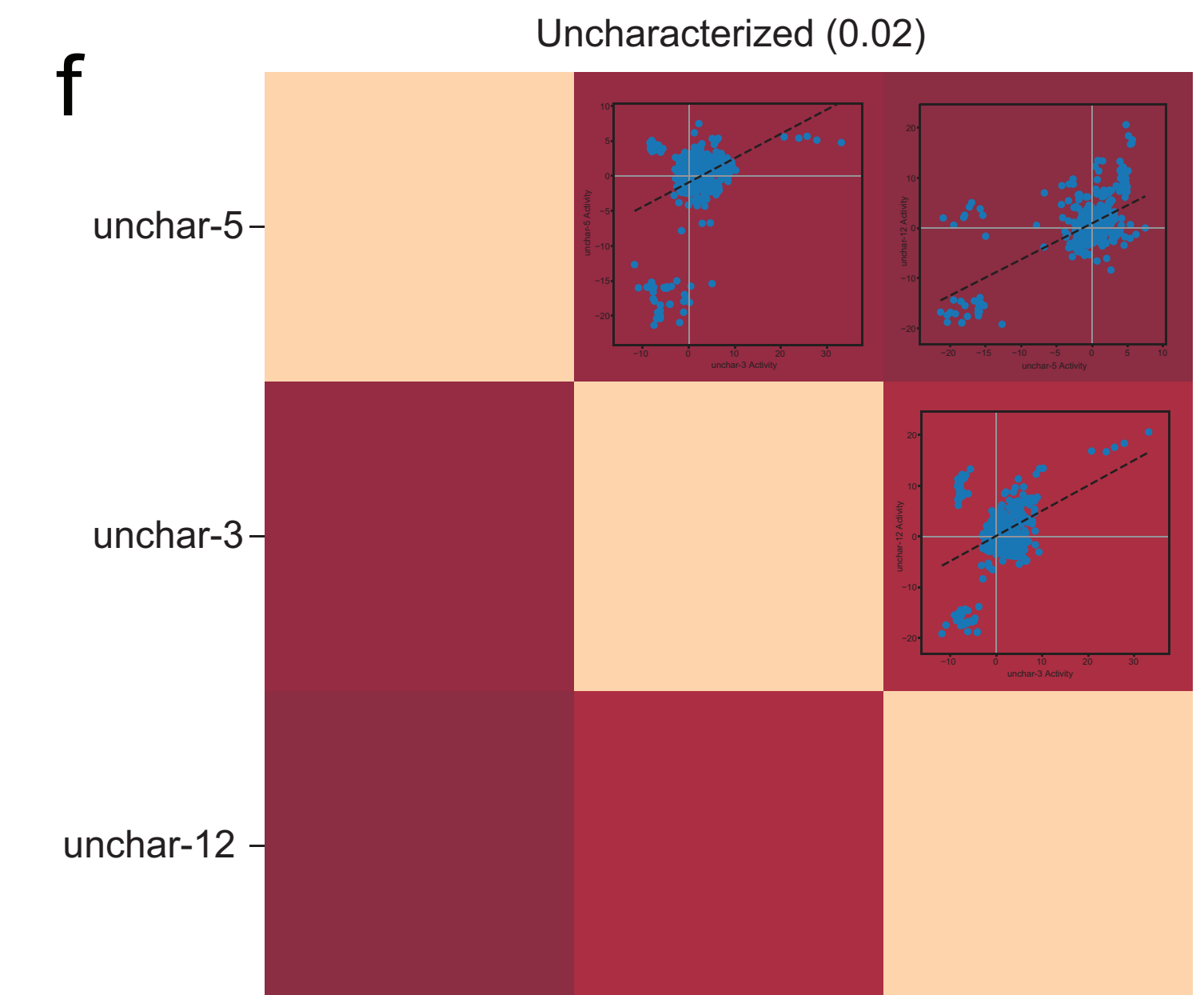

Supplement: FIG S6 [file msystems.00467-22-s0008.pdf]
