# Supplementary material for: Pan-Genome Analysis of Transcriptional Regulation in Six Salmonella enterica Serovar Typhimurium Strains Reveals Their Different Regulatory Structures
Source: mSystems. 2022 Nov 1;7(6):e00467-22. doi: 10.1128/msystems.00467-22 (PMC9764980; doi:10.1128/msystems.00467-22)
Supplement: TEXT S1 [file msystems.00467-22-s0009.docx]

**Supplementary Materials**

Pan-genome analysis of transcriptional regulation in six *Salmonella* Typhimurium strains reveals their different regulatory structures

**Authors:** Yuan Yuan, Yara Seif, Kevin Rychel, Reo Yoo, Siddharth Chauhan, Saugat Poudel, Tahani Al-bulushi, Bernhard O. Palsson, Anand Sastry

**Supplementary Notes** **2**

Supplementary Note 1: Naming of the strain 14028S 2

Supplementary Note 2: Core iModulons characterization 2

Supplementary Note 3: Tartrate utilization 2

Supplementary Note 4: Prophage presence in different strains 2

Supplementary Note 5: Comparative studies guide investigation of stress response in related strains 3

**Supplementary References**  **4**

**Supplementary Notes**

**Supplementary Note 1: Naming of the strain 14028S**

It was documented that Typhimurium strain ATCC 14028 was once termed just as strain 14028. However, a derivative of this original strain was found to have rough colony morphology, so it was named as 14028r (r for rough)^1^. To avoid confusion, the original smooth strain was then renamed as 14028s. This record seems to suggest that these three strains are in fact the same. Looking at the phylogenetic tree, the three strains are found in the same node close to each other, confirming their relatedness. Therefore, in our downstream analysis, these three strains were combined together and named as 14028S.

**Supplementary Note 2: Core iModulons characterization**

Information from public databases such as Gene Ontology (GO)^2^ enrichments and KEGG PATHWAY Databases^3^ were used to characterize the 55 non-regulatory core iModulons. These iModulons represent genes with coordinated actions but no known regulon. Some gene sets encompass related biological pathways that are regulated together to achieve a biological function, while other less characterized gene sets can be targets for regulon or pathway discovery. Many of the core genome iModulons capture metabolic pathways, delineate stress responses, and compartmentalize energy production. Some iModulons are related to virulence and resistance, which are essential mechanisms for understanding and combating *Salmonella* infections.

**Supplementary Note 3: Tartrate utilization**

The name used in the original bioproject is "tartarate" and we assumed it meant tartrate^4^. We are not sure which tartrate isomer was used (L/D/meso) or the aerobicity of the experiments.

**Supplementary Note 4: Prophage presence in different strains**

Prophages can contribute to bacterial virulence and affect the phenotypic features of the bacteria. The prophage repertoire of *Salmonella* is diverse, and each strain tends to have its own unique set of prophages. ICA decomposition of the individual strain datasets extracted prophage-related iModulons, indicative of the existence of certain prophages in each strain. For example, the Fels-1 and Fels-2 prophage-related iModulons are only found in LT2^5^. Both iModulons have high activity for sample surviving on tomato roots, a nutrient-limited and stressful environment. A hypothesis has been proposed that states that these two phages are lost in the virulent strains^5^. Genes related to the most common prophages to *Salmonella* Typhimurium Gifsy-1 and Gifsy-2 prophages are found across all strains except UK-1 and D23580 and the latter inactivates these two prophages^6^. We also discovered a Gifsy-3 like iModulon in strain 14028S that shows high correlation with the Gifsy-1 iModulon in strain SL1344. The correlated expression patterns may contribute to the similar immunity module of these two prophages^7^. Moreover, it was observed that the prophage iModulons in all strains except LT2 and D23580 carry part of the ST64B prophage. This phage is inactivated in strain D23580 but is completely absent in strain LT2. ST64B codes for the effector SseK3 and it was proposed that the presence of this phage enhances *Salmonella* survival in the blood^8,9^. In strain 14028S, the ST64B iModulon is activated under the treatment of mitomycin C (MMC), and in strain SL1344, it has high activities under SPI-2 inducing conditions, suggesting this phage is closely related to persistence and virulence. Our iModulon results are highly consistent with the documented existence of prophages in the studied strains. A deeper examination can reveal the difference in iModulon contents for the same prophage. Moreover, the activities of the iModulons serve to help understand transcriptional activation and regulation of prophage genes.

**Supplementary Note 5: Comparative studies guide investigation of stress response in related strains**

We examined a previous study that investigated the global gene expression differences between D23580 (ST313 isolate) and its ancestral strain ST4/74 (ST19 isolate) under infection-related conditions (PRJNA490148)^10^. The genomes of these two strains are 95% identical, but each strain has distinct phenotypic characteristics. Identifying the genes whose expression profile varies in the two strains may assist in explaining the phenotypic differences. Samples from both strains were cultured under the same infection-related conditions. This comparative study allows us to compare iModulon activities for the same conditions in different strains, and we discovered three uncharacterized iModulons in the core genome that displayed interesting activity patterns.

The Uncharacterized-5 iModulon consists of only two genes: *yaaY*, an uncharacterized gene, and *yccX* that codes for acylphosphatase. The Uncharacterized-12 iModulon is quite large and contains 49 genes. While there are genes related to virulence (*ssrA/ssr*B two component system) and fimbriae (*fimY* and *safB*), the majority of the genes in this iModulon code for putative proteins spanning a variety of functional categories (**Supplementary Figure 7a**). Although the functions of these two iModulons still remain obscure, their activities strongly indicate that they are related to ST4/74-specific mechanisms. In **Supplementary Figure 7b** and **6c**, the activities of the two iModulons were shown across all six strains. For samples from all the other strains, this iModulon has an activity around zero. However, for strain ST4/74, the activities for both iModulons range from -20 to 10. The small cluster of samples with low activities in ST4/74 are in a variety of conditions, so it is unclear what triggers the drop of iModulon activities.

However, looking at the Differential iModulon Activity (DIMA) plots of the bile shock samples from strain ST4/74 compared to ST4/74 samples cultured in LB and bile shock samples from strain D23580, we speculate that these two iModulons play a role in the bile response for ST4/74. (**Supplementary Figure 7d, e**). It can be seen that the Uncharacterized-5 and Uncharacterized-12, along with the Uncharacterized-3 iModulon show low activities under bile shock in strain ST4/74. Uncharacterized-3 is a large iModulon (113 genes) with no enrichment in any previously-defined regulon or pathways, but the functional category breakdown of the gene content can be found in **Supplementary Figure 8**. The activities of these three iModulons are also found to be correlated as shown in **Supplementary Figure 7f**. To our knowledge, strain ST4/74 is not particularly sensitive or resistant to bile salts compared to D23580 or other strains in this study. However, this strain was initially isolated from the bowel of a calf with Salmonellosis, so it is possible that it utilizes a special set of genes to interact with bile salts in the bowel. The exact mechanism remains to be determined, but the iModulons offer guidance to further explorations of this strain-specific response.

**Supplementary References**

1. Jarvik, T., Smillie, C., Groisman, E. A. & Ochman, H. Short-Term Signatures of Evolutionary Change in the Salmonella enterica Serovar Typhimurium 14028 Genome. *J. Bacteriol.* **192**, 560–567 (2010).

2. Gene Ontology Resource: 20 years and still GOing strong | Nucleic Acids Research | Oxford Academic. https://academic.oup.com/nar/article/47/D1/D330/5160994.

3. Kanehisa, M., Furumichi, M., Sato, Y., Ishiguro-Watanabe, M. & Tanabe, M. KEGG: integrating viruses and cellular organisms. *Nucleic Acids Res.* **49**, D545–D551 (2020).

4. MICROME_RNAseq_from_single_carbon_sources (ID 258617) - BioProject - NCBI. https://www.ncbi.nlm.nih.gov/bioproject/PRJEB4981.

5. Luo, Y. *et al.* Comparative Genome Analysis of the High Pathogenicity Salmonella Typhimurium Strain UK-1. *PLoS ONE* **7**, e40645 (2012).

6. Owen, S. V. *et al.* Characterization of the Prophage Repertoire of African Salmonella Typhimurium ST313 Reveals High Levels of Spontaneous Induction of Novel Phage BTP1. *Front. Microbiol.* **8**, 235 (2017).

7. Hiley, L., Fang, N.-X., Micalizzi, G. R. & Bates, J. Distribution of Gifsy-3 and of Variants of ST64B and Gifsy-1 Prophages amongst Salmonella enterica Serovar Typhimurium Isolates: Evidence that Combinations of Prophages Promote Clonality. *PLOS ONE* **9**, e86203 (2014).

8. Brown, N. F. *et al.* Salmonella Phage ST64B Encodes a Member of the SseK/NleB Effector Family. *PLoS ONE* **6**, e17824 (2011).

9. Herrero-Fresno, A., Leekitcharoenphon, P., Hendriksen, R. S., Olsen, J. E. & Aarestrup, F. M. Y. 2014. Analysis of the contribution of bacteriophage ST64B to in vitro virulence traits of Salmonella enterica serovar Typhimurium. *J. Med. Microbiol.* **63**, 331–342.

10. Canals, R. *et al.* Adding function to the genome of African Salmonella Typhimurium ST313 strain D23580. *PLoS Biol.* **17**, (2019).
