# Supplementary material for: Pan-Genome Analysis of Transcriptional Regulation in Six Salmonella enterica Serovar Typhimurium Strains Reveals Their Different Regulatory Structures
Source: mSystems. 2022 Nov 1;7(6):e00467-22. doi: 10.1128/msystems.00467-22 (PMC9764980; doi:10.1128/msystems.00467-22)
Supplement: FIG S3 [file msystems.00467-22-s0005.pdf]

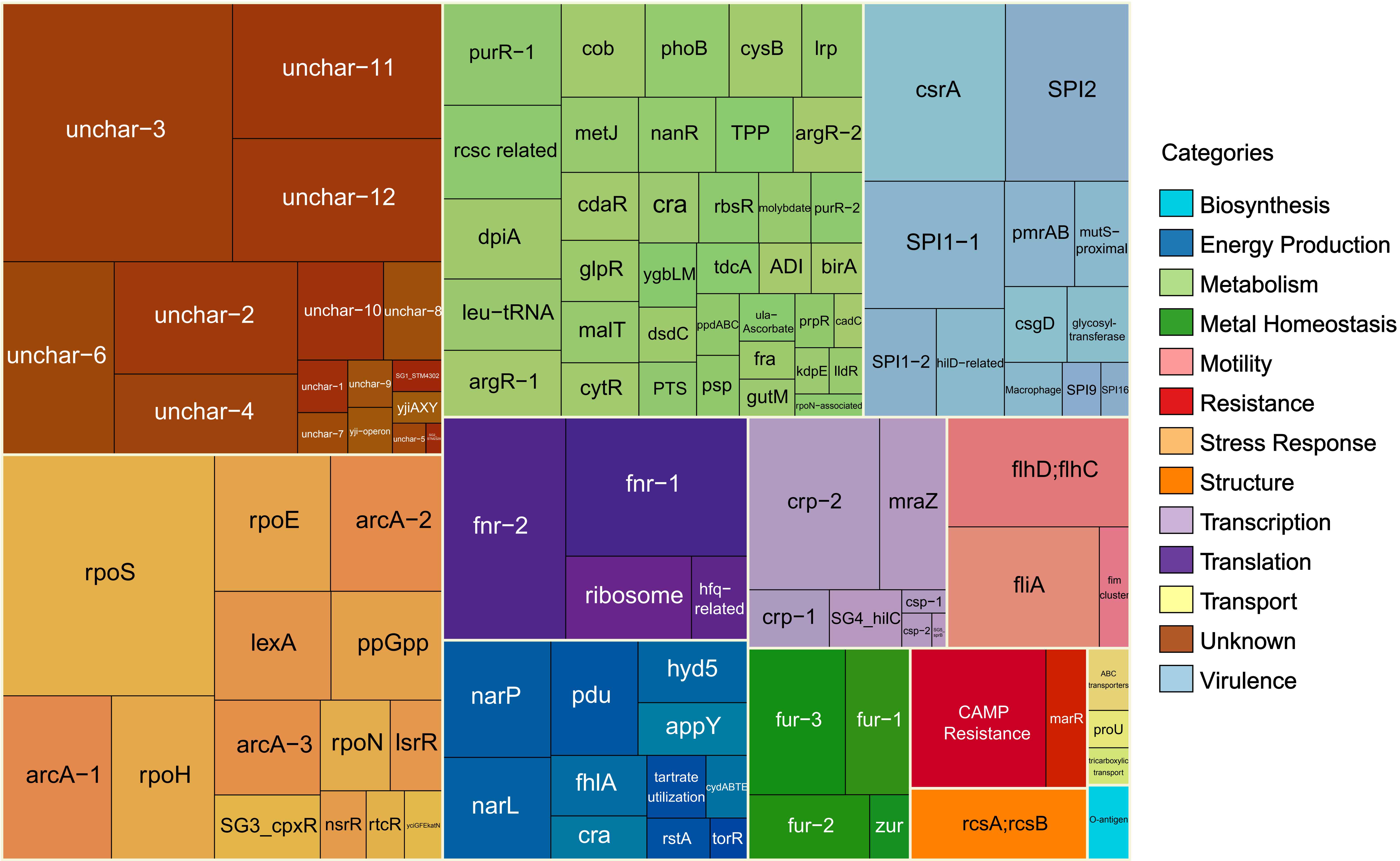
unchar−3
unchar−11
unchar−12
unchar−6
unchar−2
unchar−4
unchar−10
unchar−8
unchar−1
unchar−9
SG1_STM4302
yjiAXY
unchar−7
yji−operon
unchar−5
SG2_ STM2329
rpoS
rpoE
arcA−2
lexA
ppGpp
arcA−1
rpoH
arcA−3
rpoN
lsrR
SG3_cpxR
nsrR
rtcR
yciGFEkatN
purR−1
cob
phoB
cysB
lrp
rcsc related
metJ
nanR
TPP
argR−2
dpiA
cdaR
cra
rbsR
molybdate
purR−2
leu−tRNA
glpR
ygbLM
tdcA
ADI
birA
malT
dsdC
ppdABC
ula− Ascorbate
prpR
cadC
argR−1
fra
kdpE
lldR
cytR
PTS
psp
gutM
rpoN−associated
csrA
SPI2
SPI1−1
pmrAB
mutS− proximal
csgD
glycosyl- transferase
SPI1−2
hilD−related
Macrophage
SPI9
SPI16
fnr−2
fnr−1
ribosome
hfq− related
crp−2
mraZ
crp−1
SG4_hilC
csp−1
csp−2
SG5_ sprB
flhD;flhC
fliA
fim cluster
narP
pdu
hyd5
appY
narL
fhlA
tartrate utilization
cydABTE
cra
rstA
torR
fur−3
fur−1
fur−2
zur
CAMP Resistance
marR
ABC transporters
proU
tricarboxylic transport
rcsA;rcsB
O-antigen
Categories
Biosynthesis
Energy Production
Metabolism
Metal Homeostasis
Motility
Resistance
Stress Response
Structure
Transcription
Translation
Transport
Unknown
Virulence
